# Metabolic Remodeling in Moderate Synchronous versus Dyssynchronous Pacing-Induced Heart Failure: Integrated Metabolomics and Proteomics Study

**DOI:** 10.1371/journal.pone.0118974

**Published:** 2015-03-19

**Authors:** Junko Shibayama, Tatiana N. Yuzyuk, James Cox, Aman Makaju, Mickey Miller, Justin Lichter, Hui Li, Jane D. Leavy, Sarah Franklin, Alexey V. Zaitsev

**Affiliations:** 1 Nora Eccles Harrison Cardiovascular Research and Training Institute, University of Utah, Salt Lake City, Utah, United States of America; 2 Department of Internal Medicine, University of Utah School of Medicine, Salt Lake City, Utah, United States of America; 3 Department of Pathology, University of Utah School of Medicine, Salt Lake City, Utah, United States of America; 4 ARUP Laboratories, Salt Lake City, Utah, United States of America; 5 Metabolomics Core Research Facility, University of Utah, Salt Lake City, Utah, United States of America; 6 Department of Biochemistry, University of Utah, Salt Lake City, Utah, United States of America; 7 Department of Bioengineering, University of Utah, Salt Lake City, Utah, United States of America; Mayo Clinic, UNITED STATES

## Abstract

Heart failure (HF) is accompanied by complex alterations in myocardial energy metabolism. Up to 40% of HF patients have dyssynchronous ventricular contraction, which is an independent indicator of mortality. We hypothesized that electromechanical dyssynchrony significantly affects metabolic remodeling in the course of HF. We used a canine model of tachypacing-induced HF. Animals were paced at 200 bpm for 6 weeks either in the right atrium (synchronous HF, SHF) or in the right ventricle (dyssynchronous HF, DHF). We collected biopsies from left ventricular apex and performed comprehensive metabolic pathway analysis using multi-platform metabolomics (GC/MS; MS/MS; HPLC) and LC-MS/MS label-free proteomics. We found important differences in metabolic remodeling between SHF and DHF. As compared to Control, ATP, phosphocreatine (PCr), creatine, and PCr/ATP (prognostic indicator of mortality in HF patients) were all significantly reduced in DHF, but not SHF. In addition, the myocardial levels of carnitine (mitochondrial fatty acid carrier) and fatty acids (12:0, 14:0) were significantly reduced in DHF, but not SHF. Carnitine parmitoyltransferase I, a key regulatory enzyme of fatty acid ß-oxidation, was significantly upregulated in SHF but was not different in DHF, as compared to Control. Both SHF and DHF exhibited a reduction, but to a different degree, in creatine and the intermediates of glycolysis and the TCA cycle. In contrast to this, the enzymes of creatine kinase shuttle were upregulated, and the enzymes of glycolysis and the TCA cycle were predominantly upregulated or unchanged in both SHF and DHF. These data suggest a systemic mismatch between substrate supply and demand in pacing-induced HF. The energy deficit observed in DHF, but not in SHF, may be associated with a critical decrease in fatty acid delivery to the ß-oxidation pipeline, primarily due to a reduction in myocardial carnitine content.

## Introduction

Heart failure (HF) is a leading cause of mortality in developed countries with a current prevalence of over 5.8 million in the United States and over 23 million worldwide [1]. HF is characterized by alterations in cardiac hemodynamics secondary to depressed contractile function. Initial remodeling of electrophysiology, structure, and metabolism probably compensates for defects produced by HF. However, maladaptive changes may be associated with progression of HF phenotype, arrhythmia, and sudden cardiac death. Electromechanical dyssynchrony confers independent risk for worsened morbidity and mortality in HF [2]. Nearly half of patients with dilated cardiomyopathy have interventricular conduction delays, leading to the development of uncoordinated contraction. Dyssynchrony provides mechanical stress, primarily due to exaggerated stretch in early systole and late systolic contraction against increased afterload. Recent study demonstrated that remarkable remodeling in the transverse tubular system (t-system), which is responsible for sufficient excitation-contraction coupling, occurred in dyssynchronous HF (DHF) while synchronously contracting failing hearts had subcellular structures similar to normal hearts [3]. Thus, dyssynchrony is not only a predictor of mortality but also independently contributes to myocardial structural remodeling. Although a recent study revealed mitochondrial uncoupling and the decrease of oxidative phosphorylation efficiency in a canine model of DHF [4], it remains unknown whether dyssynchrony independently contributes to remodeling of cardiac metabolism and energetics.

In the present study we integrated metabolomic profiling of myocardial tissue and plasma with proteomic profiling for analysis of metabolic remodeling in well-established canine model of rapid pacing-induced HF [3–10]. In one group of animals the pacing was applied to the right atrium providing synchronous mode of ventricular activation (SHF model). In another group of animals pacing was applied to the right ventricle, leading to dyssynchronous ventricular activation (DHF model), which mimics the left bundle branch block, a common complication of HF in human patients. Comparison to previous studies suggested that our SHF and DHF models represented a relatively early or moderate degree of HF progression. We found energy decompensation in DHF model, whereas in SHF the energy profile remained normal. In addition, the myocardial levels of two fatty acids and carnitine, the important carrier molecule involved in fatty acid transport to mitochondria, were significantly reduced in DHF, but not in SHF. An interesting finding in both SHF and DHF models was the apparent conflict between a decrease in tissue levels of many metabolic substrates and intermediates (including intermediates of the TCA cycle and glycolysis) and upregulation of many catabolic enzymes. These data suggest a mismatch between substrate supply and demand, aggravated by cellular carnitine deficiency, as the major mechanism of energetic decompensation in dyssynchronous pacing-induced HF.

## Materials and Methods

### Ethics Statement

The experimental protocol conformed to the Guide for the Care and Use of Laboratory Animals (The National Academy Press, 8^th^ edition, 2010) and was approved by University of Utah IACUC.

### Animal instrumentation and sample collection

The general methods of tachypacing-induced canine HF model are described elsewhere [3,6–8,10]. In this study, young purpose-bred (hound mix) dogs of either sex (age, 12.8 ± 0.8 months; weight, 25.6 ± 0.5 kg) were used. Animals were obtained from an USDA Class A vendor Covance (Princeton, NJ, USA) and through the United States NIH pilot project for the *NIH Plan to Transition from the use of USDA Class B Dogs to Other Legal Sources* (http://grants.nih.gov/grants/guide/notice-files/NOT-OD-11-055.html). Animals were checked four times a day to make sure that they were kept in a clean and warm room and provided with enough water and food. The animals were instrumented with implantable pacemaker (Adapta ADDR01, Medtronic Inc., Minneapolis, MN) and intravenous pacing leads and were paced at 200 beats per minute either in the right atrium (SHF; n = 6) or right ventricular free wall (DHF; n- = 9) for 6 weeks. For both implantation and terminal study animals were induced with propofol (0.1mg/kg, IV) and maintained under deep anesthesia by inhalation of isoflurane (~2% in pure oxygen). After pacemaker implantation the animals were monitored twice a day for 7–10 days until complete wound healing and full recovery from the surgery was assured. Prophylactic antibiotics were given orally as follows: enrofloxacine- 10 mg/kg once a day for 7–10 days, amoxicillin- 10 mg/kg twice a day for 7–10 days. Narcotic analgesic Buprenorphine, 0.01–0.02 mg/kg, was administered immediately after surgery, the evening following surgery, and the morning of the first post-operation day. Additional doses of Bupernorphine were given 6–12 hours apart as judged necessary by the veterinary staff, if the animal appeared to be in post-operative pain. If necessary, a neck collar was used to prevent the animals from touching the generator implant site until healing was complete. Pacing was turned on after complete recovery from implantation surgery (about 1 week) and turned off one day before the terminal study. At terminal study, ECG was recorded and left ventricular (LV) pressure was measured in anesthetized animals using Millar pressure catheter (SPR-340, Millar, Inc., Houston, TX). After that the chest was opened through median sternotomy and cardiac biopsies (~ 100 mg) were collected from the epicardial surface of the LV apex. The tissue samples were immediately frozen in liquid nitrogen and stored at-80°C until used for various analyses. At the end of the terminal surgery, under deep anesthesia the animals were euthanized by removing the heart. The harvested heart was used for ex-vivo studies concerning the remodeling of excitation-contraction coupling in HF and not directly related to this study.

Blood samples (10 ml) were collected with heparin from venous catheter in conscious dogs before anesthesia. In Control dogs, blood was collected only once, before the terminal surgery during which the LV tissue sample was obtained. In animals used in both SHF and DHF groups, blood was collected twice. The first sample was collected before implantation of the pacemaker (*SHF-pre* and *DHF-pre*). The second sample was collected before the terminal study after 6 weeks of tachypacing (*SHF-post* and *DHF-post*). This makes total of 5 blood sample groups. The collected blood was then quickly centrifuged to remove red blood cells, and plasma was separated and stored at-80°C until used for metabolomic analysis.

### Metabolomic analysis

Myocardial tissue samples were analyzed using gas chromatography/mass spectrometry (GC/MS), tandem mass spectrometry (MS/MS), and high-pressure liquid chromatography (HPLC) as described in detail below. Plasma samples were analyzed using only GC/MS and MS/MS. HPLC analysis was not applied to plasma samples assuming that the plasma levels of metabolites determined by HPLC (adenine nucleotides, creatine, phosphocreatine (PCr), and NAD^+^) are of low relevance for the pathophysiology of HF. The complete data sets for myocardial and plasma metabolomics are presented in S1 Dataset and S2 Dataset, respectively.

**High-energy phosphate profile.** ATP, ADP, AMP, PCr, creatine, and NAD^+^ levels were determined by HPLC as described in our previous work [11]. Standard stock solutions were freshly prepared on the day of experiments (ATP, ADP, AMP, PCr, creatine, and NAD, all from Sigma, St Louis, MO, USA), and dilution was made by adding mobile phase immediately before each assay, which was used as references for peaks quantification. The cocktail of these standards was run before and after actual samples to make sure that the system performance did not decline. Approximately 10 mg of frozen wet tissue was homogenized in 120 μl of 0.4 M perchloric acid using a ceramic bead tube kit (MO BIO Laboratories, Inc., Carlsbad, CA) and Bead Ruptor (Omini International, Kennesaw, GA), which provides ultra-rapid shaking. After precipitation with 1M KOH the extracts were centrifuged for 3 min at 4°C (13,000 g). The supernatant was centrifuged at 4°C twice (14,000 g). Twenty microliters of the extracted sample was manually injected to the HPLC system.

**GC/MS for unbiased metabolomics.** Untargeted screening of metabolome was performed as described in our previous publication [11]. In brief, the freeze-clamped tissue samples (~10 mg) were homogenized in the extraction solution (methanol/water (8:1) with Cambridge Isotope’s Cell Free stable isotope labeled amino-acid mix, CNLM-6696–1, and D4-succinate standards) using ceramic bead lysis described above (speed: 6.5, time: 40 sec). Those homogenates were incubated for one hour at-20°C. After centrifugation (13,000 g at 4°C for 10 min), the supernatant was centrifuged to remove large molecules. The extraction samples were dried using speed-vac (MiVac Duo, Barnstead/Genevac Inc., Gardiner, NY) overnight. All GC-MS analysis was performed with a Waters GCT Premier mass spectrometer fitted with an Agilent 6890 gas chromatograph and a Gerstel MPS2 autosampler as described in Shakoury-Elizeh et al. [12] with slight modifications. Dried samples were suspended in 40 μL of a 40 mg/mL O-methoxylamine hydrochloride (MOX) in pyridine and incubated for one hour at 30°C. To autosampler vials was added 25 μL of this solution. Ten microliters of N-methyl-N-trimethylsilyltrifluoracetamide (MSTFA) was added automatically via the autosampler and incubated for 60 minutes at 37°C with shaking. After incubation 3 μL of a fatty acid methyl ester standard solution was added via the autosampler then 1 μL of the prepared sample was injected to the gas chromatograph inlet in the split mode with the inlet temperature held at 250°C. A 10:1 split ratio was used for analysis. The gas chromatograph had an initial temperature of 95°C for one minute followed by a 40°C/min ramp to 110°C and a hold time of 2 minutes. This was followed by a second 5°C/min ramp to 250°C, a third ramp to 350°C, then a final hold time of 3 minutes. A 30 m Phenomex ZB5–5 MSi column with a 5 m long guard column was employed for chromatographic separation. Helium was used as the carrier gas at 1 mL/min. Due to the high amounts of several metabolites the samples were analyzed once more at a 10-fold dilution. Data were collected by MassLynx 4.1. Initial analysis of known metabolites was performed using QuanLynx with data transfer to Excel. Peak picking was performed using MarkerLynx with data mining performed using SIMCA-P ver. 12.0.1. The values were normalized by wet tissue weight. To monitor for instrument sensitivity prior to analysis and drift throughout the analysis quality control samples were made up of the internal standard mix at the same concentration as each sample and analyzed at the beginning, middle and end of the experiment. The internal standard D4-succinate in each sample provided a means to normalize samples and quality control, and allow for the monitoring of instrument efficiency across batches. Additionally, negative controls that contain no cardiac tissue were prepared to detect chemical contamination and false-positive peaks during the subsequent GC-MS analysis.

**MS/MS for acylcarnitines and amino acid analysis.** Quantitative measurements of carnitine and acylcarnitines were performed using MS/MS at ARUP Laboratories (Salt Lake City, UT). Briefly, 7.6 μl of tissue extract containing 0.5 mg/μl of protein in methanol or 7.6 μl of plasma were applied to 3/16” filter paper disc and dried overnight. Sample discs were then extracted with 300 μL of methanol solution containing amino acid and acylcarnitine internal standards. The extract was transferred to a 96 well tray, dried and derivatized with 50 μL of butanol-HCl at 65°C for 15 min. After derivatization samples were dried and reconstituted with 100 μL of 50:50 acetonitrile: water solution containing 0.02% of formic acid. The plate was analyzed by Flow Injection Analysis-MS/MS using a Shimadzu UFLC (Shimadzu Corp, Kyoto, Japan) autosampler and an Applied Biosystems (API-4000) MS/MS. Amino acids and acylcarnitines were analyzed in neutral loss, precursor scan and selective reaction monitoring (SRM) modes. The quantitation was performed using ChemoView 2.0.2. Quality control samples were prepared from donor blood spiked with amino acid and acylcarnitine species at known concentrations spanning the normal and abnormal ranges of these metabolites in blood. Aliquots of normal and abnormal controls prepared in such manner were analyzed in parallel with all the test samples. Negative controls were prepared without cardiac tissue to monitor for carry-over from samples and detect false-positive peaks.

**Biochemical assay.** The myocardial level of lactate was quantified using a colorimetric/fluorometric assay kit (Cat# K607–100, BioVision Inc., Mountain View, CA). Briefly, the freeze-clamped tissue samples (~20 mg) were homogenized in the Lactate Assay Buffer using a ceramic bead tube kit and the Bead Ruptor described above (speed: 6.5, time: 40 sec). The lysates were then deproteinized using a spin filter (10-kDa molecular weight, Cat# 1997, BioVision Inc., Mountain View, CA) to remove enzymes, such as lactate dehydrogenase. The filtered samples (5–10 μL) were directly used for the biochemical assay, and lactate was measured with fluorescence (at Ex/Em = 535/587 nm).

### Proteomic analysis

**Sample preparation.** Protein extraction from dog heart tissue was carried out by lysing the tissue in lysis buffer (0.1 M Tris/HCL pH 7.6, 4% SDS, 0.1M DTT) and then sonicating the samples with a tip sonicator for 3 cycles of 10 seconds and incubating at 95°C for 10 minutes. The lysate was then centrifuged at 16,000 g for 15 minutes to remove any insoluble material. Lysate (200 μl) from each sample was loaded into a Vivacon 500 filter units, concentrated (10-fold) at 13000 g and then washed three times with 100 μL of UA buffer (8M Urea, 0.1M Tris/HCl pH 8.5). The concentrate was mixed with 100 μL of 50 mM iodoacetamide in UA buffer and incubated in darkness at room temperature for 20 min followed by centrifugation for 15 min (13,000g). The concentrate was then washed twice with 100 μL of UA buffer followed by two washes with 100 μL of 50 mM ammonium bicarbonate. Approximately 400 μg of protein from each sample was mixed with trypsin (1:80) in the filter and incubated overnight at 37°C. The peptides were then eluted with 50 mM ammonium bicarbonate and acidified with 1% formic acid.

**LC-MS/MS Analysis.** Proteomic analysis was carried out using a liquid chromatography/tandem mass-spectrometry (LC-MS/MS) system. Samples were diluted in 50 μl of 0.1% formic acid, and the peptides were separated on a 15 cm long, 75 μm ID reversed-phase PicoFrit column packed with 3 μm Reprosil-Pur C-18 resin, using a 210-min (from 5–30% acylcarnitine in 180 min and 30–45% acylcarnitine in 30 min) gradient. The MS/MS analysis was carried out on an LTQ-Orbitrap Velos Pro instrument (Thermo Fisher Scientific). The MS1 scans were acquired in the Orbitrap analyzer with 30,000 resolution at m/z of 400. The AGC targets were set at 1 × 10^6^ for MS1 scans 3 × 10^4^ for MS2 scans. The 20 most intense ions in each full MS1 scan were fragmented using CID. Dynamic exclusion was enabled with repeat count of 1 and exclusion duration of 60 sec. Each biological replicate was run in duplicate for the mass spectrometry analysis to ensure consistent protein identification. A control lysate, containing BSA protein, was also run on the mass spectrometer immediately before and after the samples reported here to ensure consistent and reproducible experimental conditions. Additionally statistical analysis of the entire data set ensured that the results were consistent within each experimental condition.

**Data analysis.** Raw MS files were processed with MaxQuant version [13] 1.4.1.2, for identification against the Uniprot Canine database. The search was carried out using the Andromeda search engine built in MaxQuant. The search included variable modifications for oxidation of methionine, acetylation of protein n-termini and fixed modification of carbamidomethylation of cysteine. The mass tolerance for MS1 and MS2 searches was 20 ppm and 0.5 Da, respectively. A false discovery rate of 1% was used for both proteins and peptides and only peptides with more than 6 amino acid residues were considered for identification and quantitation. Label free quantitation was carried out in MaxQuant using 3 technical replicates for each biological sample. During label free quantitation the isotopic patterns for each feature was matched across multiple runs using peptide identification, high mass accuracy and nonlinear remapping of retention times. Data was normalized using compiled peptide intensities and pair wise peptide ratios were calculated across the runs for all unique peptides mapping to a protein and displayed as an LFQ intensity value [14]. The complete data set for myocardial proteomics is presented in S3 Dataset.

### Statistical analysis

**Fig 1:**
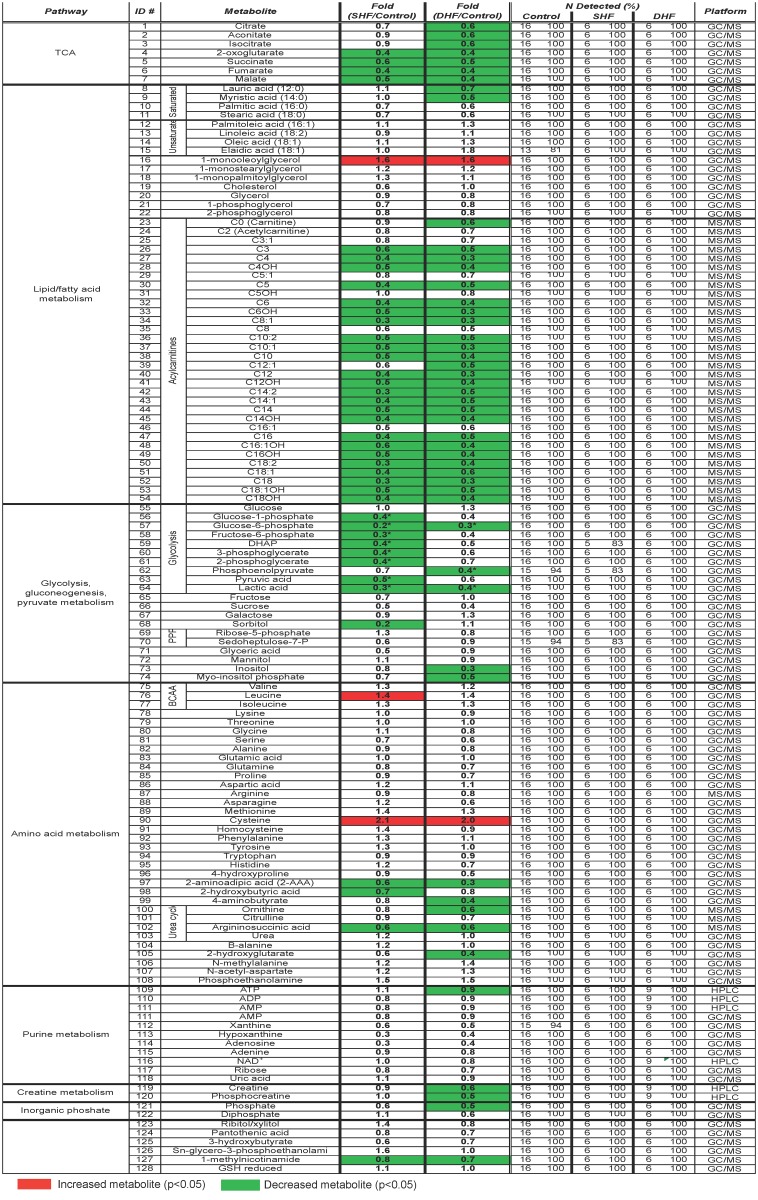
Heat map of myocardial metabolome. The data obtained by multi-platform metabolomics (GC/MS, MS/MS, HPLC) and presented as fold change in SHF and DHF as compared to Control. Green indicates a significant decrease, and read indicates a significant increase in the level of metabolite as compared to Control. BCAA: branched-chain amino acid, PPP: pentose phosphate pathway, GSH: glutathione, GC/MS: gas-chromatography/mass-spectrometry, MS/MS: tandem mass-spectrometry, HPLC: high performance liquid chromatography.

For comparisons between Control, SHF, and DHF all metabolites (except 9 intermediates of glycolytic pathway) and all detected enzymes, one-way ANOVA with Fisher’s LSD post-hoc test was applied using Metaboanalyst 2.0 software [15,16]. The nine intermediates of glycolysis (indicated in Fig. 1) were compared using non-parametric Kruskal-Wllis test with Dunn test for pairwise comparisons (XLSTAT software, Addinsoft, http://www.addinsoft.com). A value of p<0.05 was considered statistically significant. Data are given as mean ± SEM.

## Results

### Hemodynamics

The hemodynamic features of the canine SHF and DHF models have been reported previously [8,17], and the results obtained in our study are summarized in Table 1. There was no statistically significant difference in any measured parameter between SHF and DHF hearts before pacing. Both SHF and DHF models exhibited a significant reduction in left ventricular systolic pressure (LVSP) and the maximum speed of LV pressure development (dP/dt_max_). In addition, the maximum speed of LV relaxation (dP/dt_min_) was significantly decreased in both SHF and DHF hearts. LV end-diastolic pressure (LVEDP) was unchanged in SHF and moderately, but significantly elevated in DHF. Unlike our study, previous studies using essentially the same SHF and DHF pacing protocols showed a more significant and similar increase in LVEDP in both models, indicating a prominent diastolic dysfunction [6,8]. Possible reasons for this difference will be addressed in Discussion.

**Table 1:** Hemodynamics of SHF and DHF in pre- and post-tachypacing.

	SHF		DHF	
	Pre	Post	Pre	Post
LVSP (mmHg)	106.1 ± 7.5	76.4 ± 5.3*	108.6 ± 6.2	66.1 ± 4.6*
LVEDP (mmHg)	7.2 ± 2.3	11.2 ± 2.6	6.2 ± 2.7	15.2 ± 4.6*
dP/dt max (mmHg/s)	1829 ± 251	1062 ± 257*	1503 ± 121	680 ± 49*
dP/dt min (mmHg/s)	-2535 ± 308	-1310 ± 244*	-2172 ± 152	-1093 ± 139*

*:p<0.05 (pre vs. post, paired *t*-test)

### Overview of Metabolomic and Proteomic Analysis

In order to determine whether electromechanical dyssynchrony significantly affects metabolic remodeling in the course of pacing-induced HF, we performed a comprehensive analysis of metabolic pathways relevant to energy metabolism in SHF, DHF, and normal dogs using integrated metabolomics and proteomics approach.

**Metabolomics.** Combining three metabolomics platforms (GC/MS, MS/MS and HPLC, see Methods), we detected total of 128 metabolites, including 9 glycolysis intermediates, 7 TCA intermediates, 22 amino acids, 8 free fatty acids, 32 acylcarnitines, and 10 purine metabolites. The “heat map” displayed in Fig. 1 shows which of the detected metabolites were significantly different in either SHF or DHF group as compared to Control. Red color denotes a significantly elevated level of metabolites in SHF or DHF as compared to Control; green color denotes a significantly lower level than in Control. We found that in SHF 41 metabolites were significantly different from Control (decrease in 38 metabolites; increase in 3 metabolites), whereas in DHF 50 metabolites were significantly different from Control (decrease in 48 metabolites; increase in 2 metabolites). Note that in both SHF and DHF the predominant pattern of change was a *decrease* in the levels of many metabolites as compared to Control. Note also that even though the majority of changes observed in metabolite levels were similar in SHF and DHF, exclusive to DHF were significant decrease in tissue contents of carnitine, lauric and myristic fatty acids, and, more importantly, PCr and ATP (see Fig. 1).

**Fig 2:**
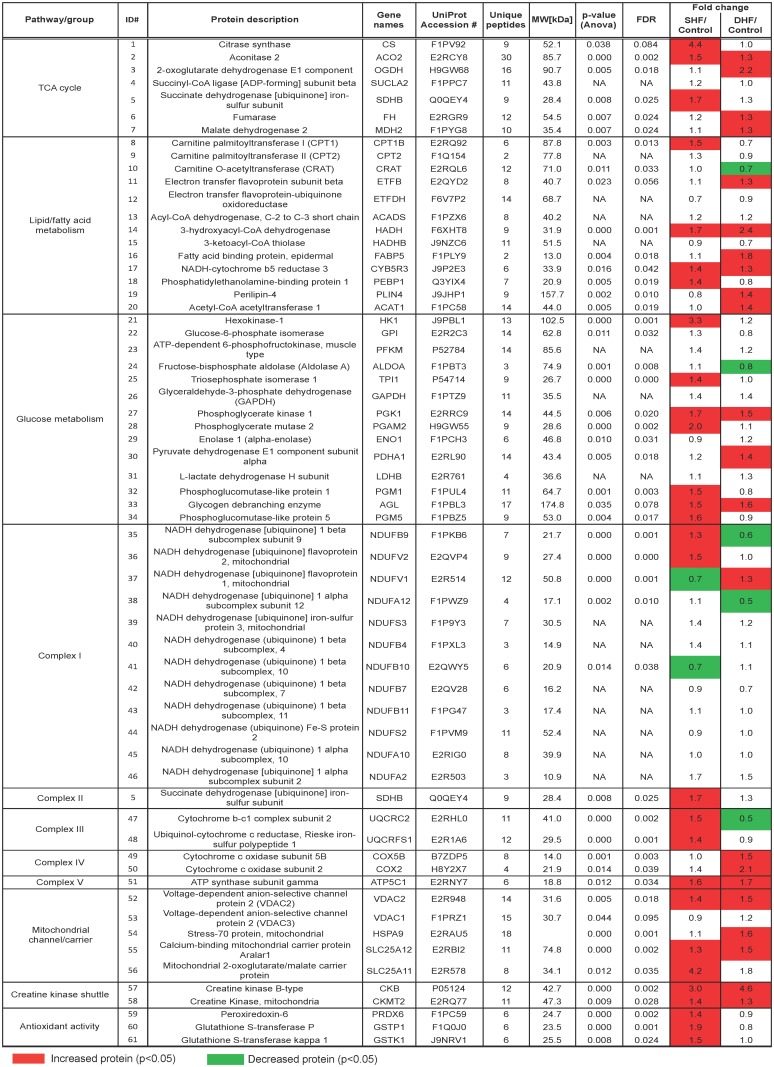
Heat map of selected metabolic proteome. Metabolism-related proteins detected by LC-MS/MS, and presented as fold change in SHF and DHF compared with those from Control. Green indicates a significant decrease, and read indicates a significant increase in the level of protein expression as compared to Control.

**Proteomics.** The LC-MS/MS-based unbiased proteomic screening of myocardial tissue extracts detected a total of 1,559 proteins. To increase the quality of relative quantification, we limited our analysis to the proteins that were detected in all 3 technical replicates from three groups, which allowed us to analyze 166 proteins as shown in S3 Dataset. Out of these 166 proteins, 61 could be mapped to pathways relevant to energy metabolism. The heat map of metabolic proteome is shown in Fig. 2. Similar to Fig. 1, red color denotes a significantly elevated level of proteins in SHF or DHF as compared to Control, whereas green color denotes a significantly lower level than in Control. It is worth to note that in contrast to metabolome (Fig. 1), which showed a pattern of predominant reduction in metabolite levels, the expression levels of metabolism-related proteins were mostly *increased or unchanged*, suggesting partial upregulation of metabolism in both models of HF. Below we will analyze pathway-specific changes in metabolome and metabolic proteome.

### Energy metabolism

**Fig 3:**
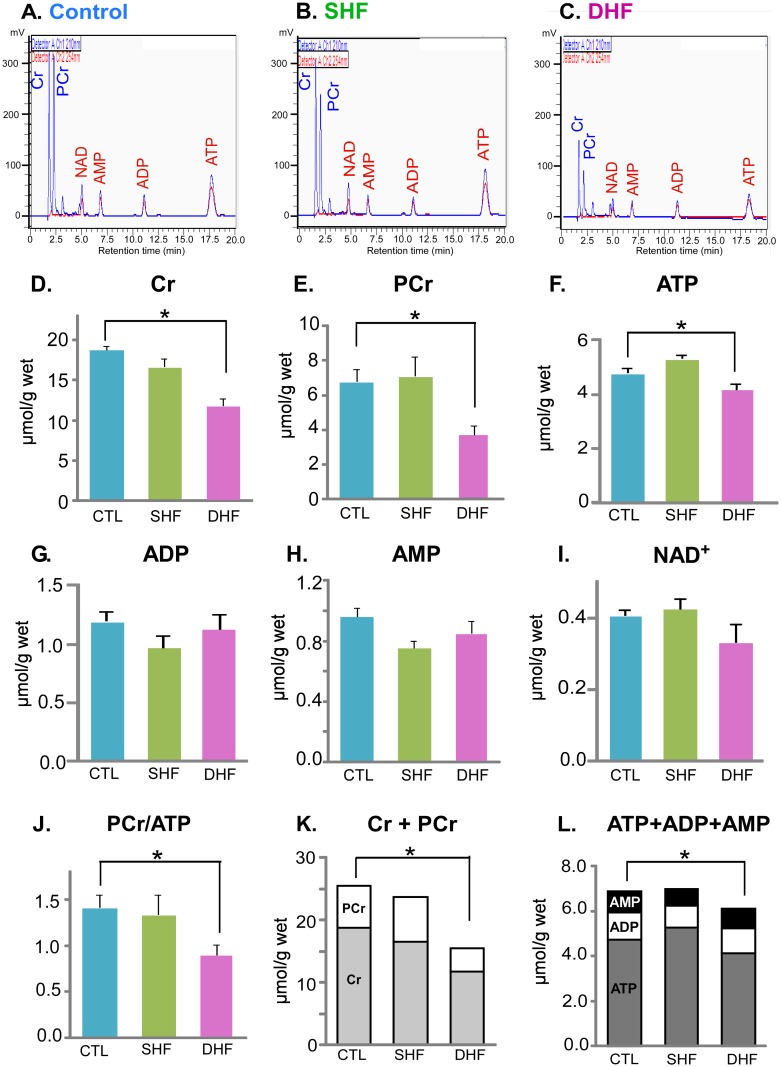
High-energy phosphates and related adenine nucleotides. Metabolites were measured by HPLC in Control, SHF and DHF hearts. **A-C.** Chromatograms of extracts from ventricular tissue in Control (A), SHF (B), and DHF (C). **D-I.** Quantitative analysis of the levels of creatine (Cr), phosphocreatine (PCr), ATP, ADP, AMP, and NAD^+^, respectively, normalized by wet tissue weight. **J.** PCr/ATP ratio. **K.** Total pool of myocardial creatine (creatine + PCr). **L.** Total pool of adenine nucleotides (ATP+ADP+AMP). *p<0.05.

Molecules involved in energy turnover (ATP, ADP, AMP, creatine, PCr, and NAD^+^) were measured in Control, SHF, and DHF animals using HPLC (Fig. 3). The upper three panels (A-C) show the representative chromatograms in Control, SHF and DHF, respectively. Other panels show the average values of the six measured metabolites and three derived parameters (total creatine pool (creatine + PCr, ∑Cr); total adenine nucleotide pool (ATP+ADP+AMP, ∑Ad); and PCr/ATP ratio). The energy profile in SHF animals was very similar to that in Control. In fact, among the 6 metabolites involved in energy turnover, only the level of creatine trended lower (p = 0.053) in SHF than in Control. The PCr/ATP ratio, ∑Cr, and ∑Ad, were not significantly different between SHF and Control hearts. In contrast, DHF group exhibited significant reduction in the levels of creatine, PCr, ATP, PCr/ATP ratio, ∑Cr, and ∑Ad. Neither SHF nor DHF showed significant alterations in the levels of ADP, AMP, and NAD^+^. We also estimated the level of free ADP (ADP_f_) using the equilibrium expression for creatine kinase [7] and computed ratios of ATP/ADP_f_ as well as ATP/ADP (see S1 Fig.). APD_f_ and ATP/ADP_f_ were not significantly different between the three groups. ATP/ADP ratio was significantly increased in SHF, but was not different in DHF as compared to Control. Lastly, the levels of the intermediates of the adenine nucleotide degradation pathway (adenine, adenosine, xanthine and hypoxanthine) detected by GS/MS trended towards reduced levels, but none of them were significantly different in SHF and DHF from Control (see Fig. 1). Summarizing above, we conclude that DHF hearts exhibited energy deficit, whereas in SHF hearts the energy balance was largely preserved.

**Fig 4:**
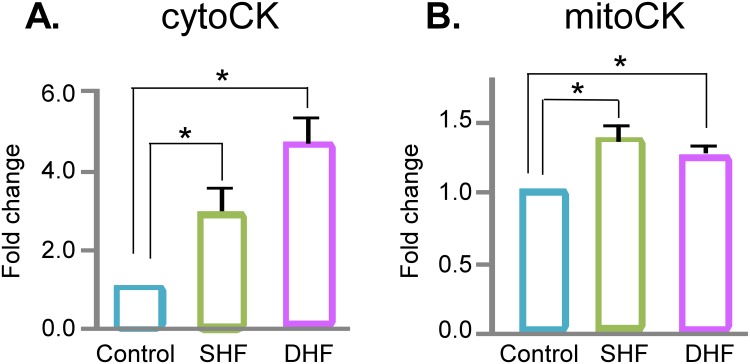
Protein expression of creatine kinase (CK) isoforms. Both cytosolic CK-B type (**A**) and mitochondrial CK (**B**) are upregulated in SHF and DHF as compared to Control. *p<0.05.

Among detected proteins those immediately related to energy production included total of 18 different subunits of the Complexes I-V of the respiratory chain. Detected subunits of Complexes I and II showed a mixed picture of upregulation and downregulation, but more subunits were upregulated in SHF, whereas more subunits were downregulated in DHF (see Fig. 2). Detected subunits of Complexes III (subunit 2, rieske iron-sulfur polypeptide 1) and IV (polypeptide 5B, subunit 2), were upregulated or unchanged in SHF and DHF. Finally, Complex V (ATP synthase) subunit gamma (gene name ATP5C1) was upregulated in both SHF and DHF. The two enzymes comprising creatine kinase (CK) shuttle system (mitochondrial and cytosolic CK), which transfer ATP produced in the mitochondrial matrix to the cytosol, were significantly upregulated in both SHF and DHF (Fig. 4). Thus, at least some enzymes involved in mitochondrial energetics and energy delivery were upregulated in both SHF and DHF.

### TCA cycle

**Fig 5:**
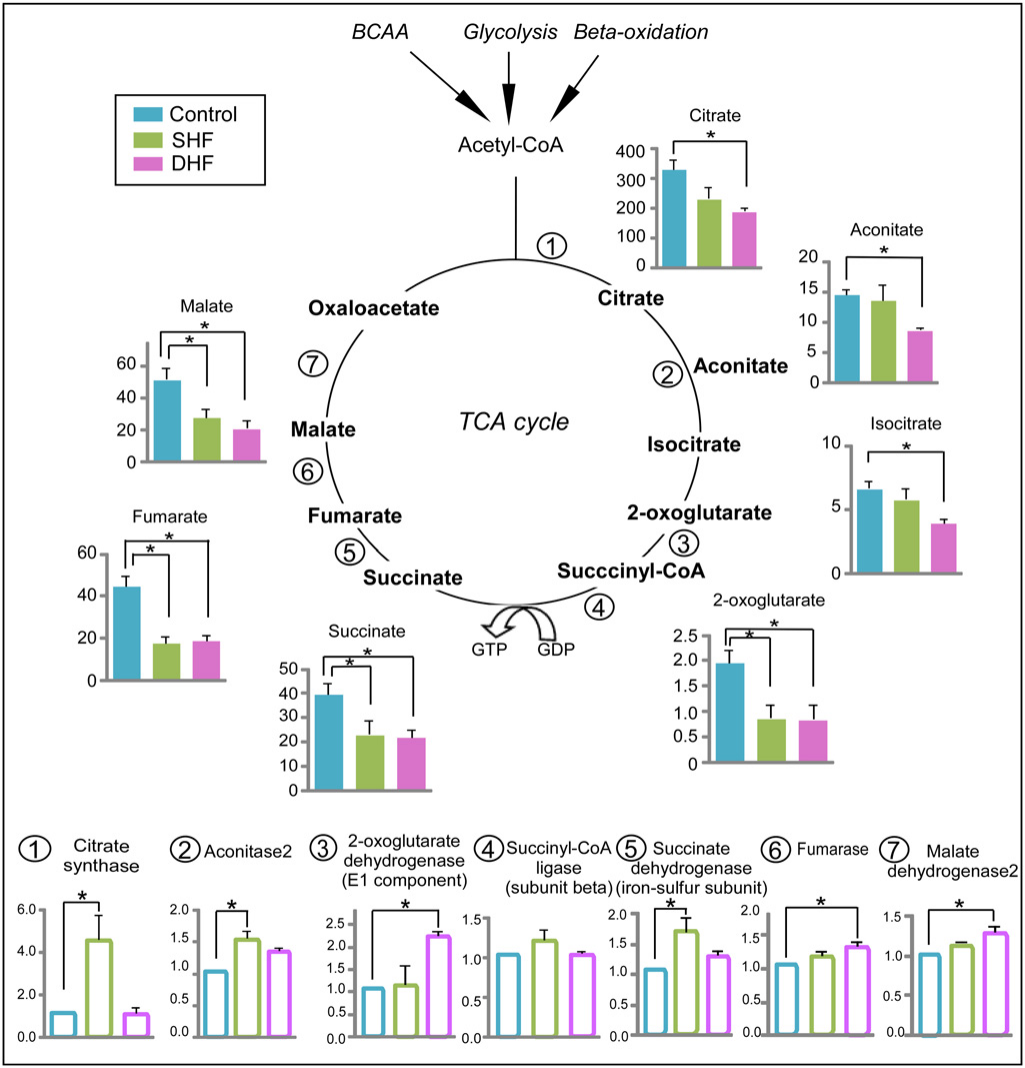
Metabolomic and proteomic profile of the TCA cycle. Data from Control, SHF, and DHF hearts. Filled bars: the TCA cycle intermediates. Open bars: the TCA cycle enzymes. Metabolome is presented in arbitrary units while proteome is presented as fold change compared to Control. BCAA: branched-chain amino acids. *P<0.05.

Modification of TCA cycle in HF has been reported previously [18,19], and the depletion of carbon flux into TCA cycle results in contractile dysfunction [20]. Using GC/MS, we were able to detect 7 TCA cycle intermediates (citrate, aconitate, isocitrate, 2-oxoglutarate, succinate, fumarate, malate). Due to the instability of molecules in our extraction method, the other two TCA intermediates (succinyl-CoA and oxaloacetate) were not detected by mass-spectrometry. As shown in Fig. 5, the levels of each detected TCA cycle intermediate decreased at least in one studied model of HF. Specifically, 4 TCA cycle intermediates (2-oxoglutarate, succinate, fumarate, malate) were significantly reduced in SHF, and all 7 detected intermediates were significantly reduced in DHF, as compared to Control. In contrast to the TCA cycle intermediates, protein levels of the TCA cycle enzymes were upregulated or unchanged in both SHF and DHF (see Fig. 5). In DHF, the expression of 3 enzymes significantly increased as compared to Control (2-oxoglutarate dehydrogenase, fumarase, and malate dehydrogenase). In SHF, citrate synthase and isocitrate dehydrogenase were significantly upregulated (~4-fold and 1.5-fold increase from Control, respectively). Thus, the integrated metabolomic and proteomic analysis revealed a specific pattern of the TCA cycle stress, in which depletion of the TCA cycle intermediates contrasted with upregulation of the TCA cycle enzymes. This pattern was evident in both HF models but was more pronounced in DHF than SHF.

### Fatty acid metabolism

**Fig 6:**
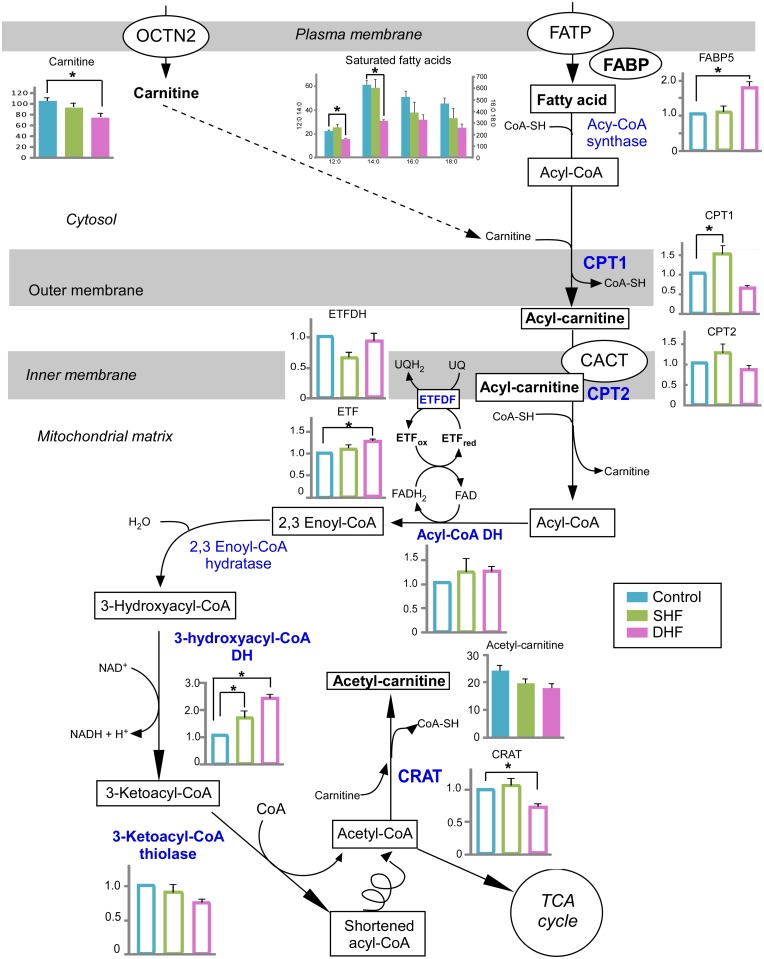
Fatty acid catabolism. A schematic overview of fatty acid catabolism is presented with the levels of detected metabolites and relevant proteins in Control, SHF, and DHF. Detected metabolites and proteins are indicated with bold font. Blue color indicates enzymes involved in fatty acid oxidation. Metabolome is presented in arbitrary units while proteome is presented as fold change compared to Control. Filled bars: metabolites, Open bars: proteins. OCTN2: organic cation transporter novel type 2, FATP: fatty acid transport protein, FABP: fatty acid binding protein, CPT1: carnitine palmitoyltransferase I, CPT2: carnitine palmitoyltransferase II, CACT: carnitine O-acetyltransferase; DH: dehydrogenase; ETF: electron-transferring flavoprotein; ETFDH: electron transfer flavoprotein-ubiquinone oxidoreductase; CRAT: carnitine O-acetyltransferase. *P<0.05.

The overview of our findings in the context of fatty acid metabolism is presented in Fig. 6. Fatty acids primarily enter a cell via fatty acid protein transporters on the cell surface, such as fatty acid translocase (FAT/CD36), tissue specific fatty acid transport proteins, and plasma membrane bound fatty acid binding protein (FABPpm, reviewed in [21]). Our proteomics screening did not detect any of the membrane-bound fatty acid transport proteins, most likely due to their tight association with the lipid bilayer. On the cytoplasmic side of the membrane, a family of fatty acid-binding proteins (FABPs) are believed to facilitate fatty acid transport inside cardiac myocytes [22]. We detected one of the proteins of this family, FABP5, which was unchanged in SHF but significantly upregulated in DHF. Metabolomic screening using GS/MS detected total of 4 saturated and 4 unsaturated fatty acids in ventricular myocardium (see Fig. 1). Among those, unsaturated fatty acids were not significantly different between Control and either of the HF models, although there was a trend towards their increase in DHF (see Fig. 1). Among saturated fatty acids, none were significantly different from Control in SHF, but the tissue levels of lauric (12:0) and mystiric (14:0) acids were significantly reduced in DHF as compared to Control. The levels of palmitic (16:0) and stearic (18:0) acids tended to decrease in both SHF and DHF, but in neither case the difference reached statistical significance (Fig. 6). Plasma levels of all FAs that were detected by GC/MS were not different between Control, SHF, and DHF (data not shown).

**Carnitine shuttle system.** In the first step of fatty acid catabolism, free fatty acids are activated by forming fatty acyl-CoAs, which are subsequently imported into the mitochondrial matrix via the carnitine shuttle system. Carnitine is required for long-chain fatty acids to cross the inner mitochondrial membrane. Carnitine-palmitoyl transferase I (CPT1) located in the mitochondrial outer membrane forms fatty acylcarnitine, which is translocated into mitochondrial matrix by the enzyme carnitine-acylcarnitine translocase (CACT) in exchange for free carnitine. Carnitine-palmitoyl transferase II (CPT2) located on the mitochondrial inner membrane converts fatty acylcarnitine back to fatty acyl-CoA, releasing the carnitine. Lastly, carnitine is shuttled back across the inner mitochondrial membrane by CACT (see Fig. 6).

**Fig 7:**
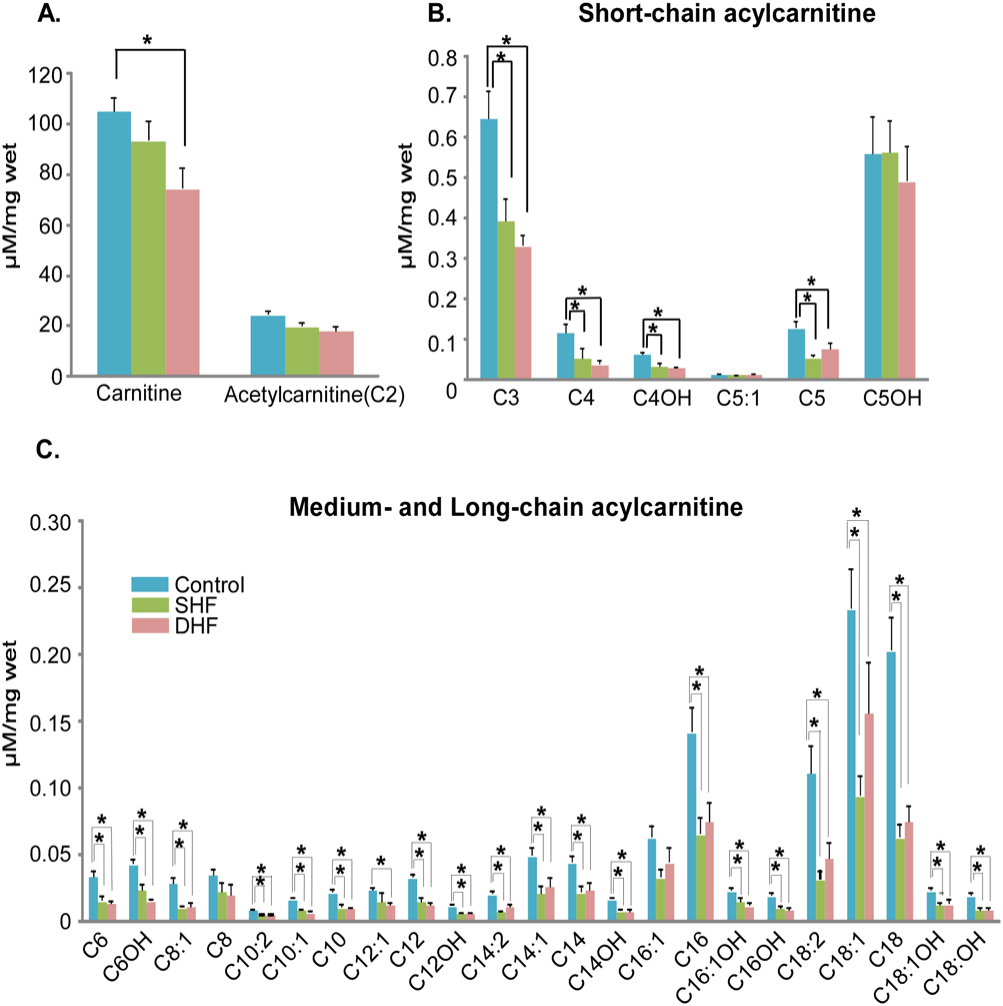
Quantitative analysis of myocardial carnitine and acylcarnitines. Majority of acylcarnitines were significantly reduced in both SHF and DHF as compared to Control (see also S3 Fig. for the levels of plasma acylcarnitines). P*<0.05

The levels of myocardial free carnitine decreased in both SHF and DHF, but we found significant difference only between Control and DHF. We also observed a remarkable reduction in short-, medium-, and long-chain acylcarnitines both in SHF and DHF. Among 30 acylcarnitines that were analyzed, 21 acylcarnitines in SHF and 24 acylcarnitines in DHF were significantly lower than those in normal hearts (Fig. 7).

We also measured the levels of acylcarnitines in plasma samples, which were collected before (pre) and after (post) tachypacing from the same animals that were used for myocardial metabolomic and proteomic analysis of myocardium after tachypacing. The fold changes of acylcarnitines between pre- and post-paced animals are shown in S2 Fig. In contrast to myocardial acylcarnitines, the plasma acylcarnitines were elevated in both SHF and DHF hearts after 6 weeks of pacing. Thus, we observed a *decrease* of myocardial acylcarnitines in parallel with and an *increase* in plasma acylcarnitines in both HF models. However, only in the DHF model the reduction in the total pool of myocardial carnitine and elevation of the total pool of plasma carnitine were statistically significant (see S3 Fig.).

CPT1 is considered to be the rate-limiting step of fatty acid catabolism [21]. It has been reported that CPT1 was downregulated in tachypacing-induced HF [10]. In our study, the protein level of CPT1 was significantly increased in SHF and trended to decrease in DHF, but the latter difference did not reach statistical significance. The protein levels of CPT2 trended to increase in SHF and decrease in DHF, but in both models these changes were not statistically significant.

**Fatty acid ß-oxidation (FAO).** ß-oxidation of saturated fatty acids requires 4 enzymatic reactions catalyzed by acyl-CoA dehydrogenase, 2,3 enoyl CoA hydratase, 3-hydroxyacyl CoA dehydrogenase, and 3-ketoacyl-CoA thiolase. Our proteomic screening detected acyl-CoA dehydrogenase (short-chain), 3-hydroxyacyl CoA dehydrogenase (short- and medium-chain), and 3-ketoacyl-CoA thiolase (long-chain, ß-subunit of trifunctional protein). Among these proteins, 3-ketoacyl-CoA thiolase was significantly upregulated in both HF models, this increase being more pronounced in DHF than SHF. In addition, the expression level of electron transfer flavoprotein (ETF), which, together with ETF dehydrogenase (ETFDH) transfers high energy electrons from FADH_2_ to the respiratory chain, was significantly increased in DHF, although the expression level of ETFDH was similar between Control and DHF. Carnitine O-acetyltransferase (CRAT), which plays a role in transferring excess acetyl-CoA from mitochondria to cytosol by forming acetyl-carnitine with carnitine, was significantly decreased in DHF, but not in SHF.

Overall, these findings suggest upregulation of FAO in SHF, whereas changes occurring in DHF are mixed, with some enzymes upregulated and some downregulated.

### Glycolysis

**Fig 8:**
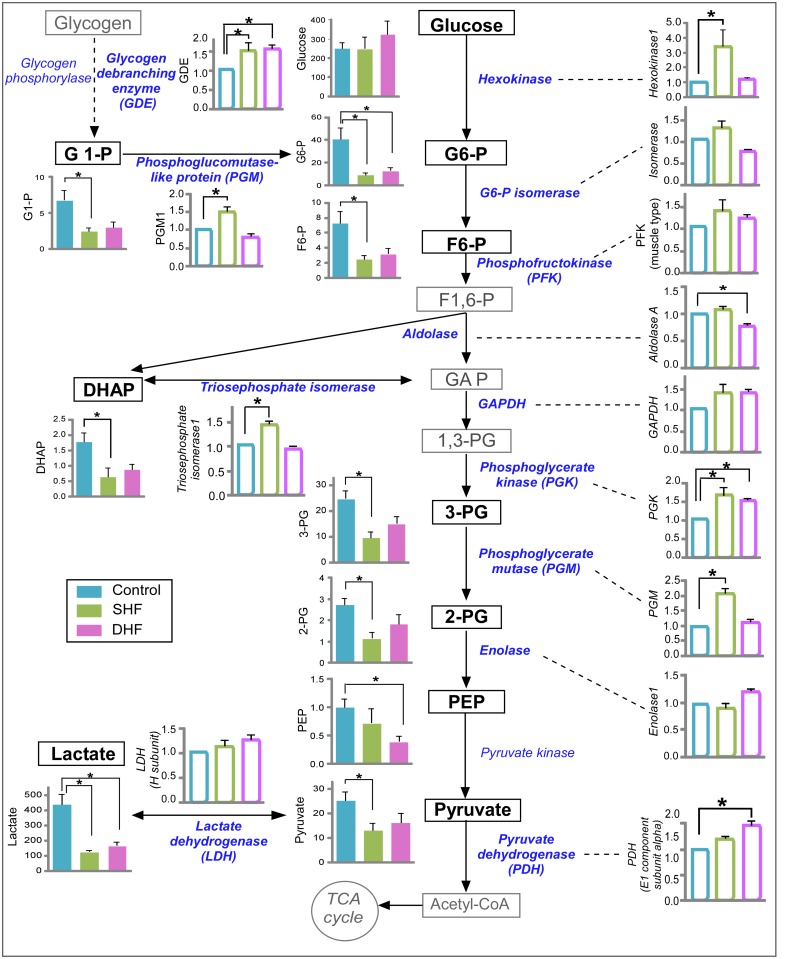
Metabolomic and proteomic profile of glucose metabolism. Data from Control, SHF, and DHF hearts. Detected metabolites and enzymes are indicated with bold font. Metabolome is presented in arbitrary units while proteome is presented as fold change compared to Control. Filled bars: metabolites. Open bars: proteins. G1-P: glucose 1-phosphate, G6-P: glucose 6-phosphate, F6-P: fructose 6-phosphate, F1,6-P: fructose 1,6-bisphosphate, GAP: glycealdehydo 3-phosphate, DHAP: dihydroxyacetone phosphate, 1,3-PG: 1,3-bisphosphoglycerate, 3-PG: 3-phosphoglycerate, 2-PG: 2-phosphoglycerate, PEP: phosphoenolpyruvate. P*<0.05.

It is known that HF leads to switching in substrate preference from fatty acids to glucose [21]. The metabolomic profile of glucose pathway with its enzyme expression is shown in Fig. 8. The myocardial levels of glucose were not different between Control, SHF, and DHF. However, the average values of all the intermediates of glycolysis pathway that were detected by GC/MS decreased in both models of HF.

Many glycolytic intermediates exhibited a large variation between measurements taken in different hearts, especially in the Control group. Of interest, some of the glycolytic intermediates showed a high degree of mutual correlation. For example, if one heart had a small value of glucose 6-phosphate, it also had a small value of fructose 6-phosphate, and, conversely, if it had a large value of glucose 6-phosphate, it also had a large value of fructose 6-phosphate. Whereas correlation was consistently strong between glucose 6-phosphate, glucose-1-phosphate and fructose 6-phosphate (in all pairwise combinations), the pattern of correlation between other intermediates was generally not as strong and varied between the Control, SHF, and DHF groups (see S4 Dataset). We believe that the large dispersion between individual measurements of glycolytic intermediates is not due to error of measurement, but rather reflects glycolytic oscillations [23,24]; this would explain the correlation between some glycolytic intermediates. An extended analysis of glycolytic intermediates is presented in S1 Text, S4 Dataset, and S4 Fig. and S5 Fig. Statistical analysis using a non-parametric statistical test showed that out of 9 detected intermediates 8 were significantly reduced in SHF and 3 were significantly reduced in DHF as compared to Control (see Fig. 8). Importantly, in both groups the levels of glucose 6-phosphate and lactate were significantly lower than in Control. Since the decrease of tissue lactate in HF has not been previously reported, we performed quantitative analysis of myocardial lactate in the DHF group using a biochemical assay. Consistent with our metabolomic measurement, we found that myocardial lactate was significantly decreased in DHF as compared to Control (see S6 Fig.).

The proteomic data showed that the expression of 7 glycolytic enzymes (hexokinase 1, triose-phosphate isomerase, phosphoglycerate kinase, and phosphoglycerate mutase, phosphoglucomutase isoforms 1 and 5, and glycogen debranching protein) significantly increased in SHF, whereas 3 enzymes (phosphoglycerate kinase, pyruvate dehydrogenase, and glycogen debranching protein) were significantly increased in DHF. The key regulatory enzymes of glycolysis (glyceraldehyde-3-phosphate dehydrogenase and phosphofructokinase) tended to increase in SHF and DHF, but the difference did not reach statistical significance. Overall, the comparison of glycolysis intermediates and enzymes revealed a pattern somewhat similar that observed in the TCA cycle: that is, a contract between decreased levels of metabolites versus increased or unchanged levels of catabolic enzymes.

### Plasma metabolome

Total of 110 metabolites were detected in plasma, including glycolysis and TCA intermediates, amino acids, free fatty acids, and acylcarnitines. Heat map of plasma metabolomics is shown in S7 Fig., presented as fold change in SHF and DHF animals after 6 weeks of pacing (“post”) as compared to those from the same animals before pacing (“pre”). A complete data set of plasma metabolome can be found in S2 Dataset. In addition to increased levels of free carnitine and acylcarnitines mentioned above in relation to fatty acid metabolism (see S2 Fig.), noteworthy observations included unchanged levels of blood glucose (also see S8 Fig., Panel B) and saturated fatty acids in both models of HF.

### Miscellaneous observations

Additional noteworthy observations are presented and discussed in S1 Text.

## Discussion

In this study we sought to obtain a comprehensive picture of metabolome and metabolic proteome in two variants of tachypacing-induced canine HF model, synchronous (SHF) and dyssynchronous (DHF). These two models were previously characterized in terms of regional expression of key proteins involved with stress response, muscle mechanics, and electrophysiology [8]; sarcomere shortening and calcium responses to β-adrenergic receptor stimulation [25]; and regional remodeling of the t-system [3] and the sarcomeric cytoskeleton [6]. Those studies showed that SHF and DHF had similar levels of global LV dysfunction and depression of sarcomere shortening, but were different with respect to regional variability in protein expression and some features of structural sarcomere remodeling. The importance of understanding the difference between SHF and DHF stems from the fact that electromechanical dyssynchrony occurs in approximately 40% of patients suffering from HF and has been shown to be an independent predictor of mortality and sudden cardiac death [2]. However, it remained unknown whether SHF and DHF models follow the same or different paths of metabolic remodeling.

Our approach to address this problem was to combine different platforms available to us in order to collect as much information as possible from the same tissue samples. For the purpose of metabolic analysis, we combined unbiased GC/MS metabolomics with targeted platforms for quantitative analysis of energy metabolism (using HPLC) and acylcarnitines (using MS/MS). The importance of metabolomic profiling is that it presents a systems view of interconnected metabolic pathways and may reveal emergent properties of the metabolic system as a whole, which are not detectable or not interpretable when analysis is focused only on a smaller subsystem. The inherent limitation of metabolomics is that in order to fully describe the function of the metabolic system the mass ratios of all metabolites need to be known, as well all inputs and outputs to the system. At present, this is not practically achievable. Currently, the metabolomic analysis operates in terms of a relative increase or decrease of each detected metabolite between the Control and the test group(s). The problem is that the change in the level of any metabolite may be due to a change in the input, or the output, or both. Most of the time, lacking full information about every input and output, the interpretation of the direction of change is equivocal. In order to increase the interpretability of the results, we enhanced tissue metabolomics in two ways, by a parallel analysis of plasma metabolome and the analysis of metabolic proteome. The integration of the three sets of data revealed an unexpected “state of conflict” between the changes in cardiac metabolites (decrease or no change), plasma metabolites (increase or no change) and metabolic enzymes (increase or no change, see Figs. 1, 2 and S7 Fig.). Below we will discuss the patterns of change in different metabolic pathways/subsystems and then will try to reconstruct a holistic picture of metabolic remodeling in SHF and DHF models.

### Energy metabolism

In the myocardium from failing human hearts and animal models of severe HF, [ATP] is ~30% lower than in normal myocardium. The fall in [ATP] occurs in different species, different experimental HF models and due to various etiologies of the disease in human patients [26]. A decrease in the [PCr] may precede a dramatic fall in [ATP], and the decrease in PCr/ATP ratio is an independent predictor of mortality in HF patients [27].

SHF and DHF hearts in our study had similar level of systolic dysfunction. Diastolic dysfunction was not detectable in SHF and moderate in DHF (Table 1), which is in contrast to a pronounced diastolic dysfunction previously reported in similar canine SHF and DHF models [8,25] and probably places our SHF and DHF models at an earlier stage of HF progression. Although the hemodynamic outcomes were similar in SHF and DHF, in terms of energy metabolism the two models were principally different, as SHF maintained normal energy profile whereas DHF showed clear signs of energetic decompensation. Exclusive to DHF were significant decreases in creatine, ATP, PCr/ATP ratio, as well as the total creatine pool (∑Cr) and the total adenine nucleotide pool (∑Ad, see Fig. 3).

Several prior studies addressed changes in energy metabolism associated with experimental HF [7,28,29]. However, the direct comparison of prior work with our data is complicated due to differences in assays used, the actual subset of metabolites which were quantified, and the approaches used for normalization (tissue wet weight, tissue dry weight, or total protein). Additional confounding factors include the age of dogs (often not known or not reported) and the anesthetic used during sample collection. Lastly, variability in the HF model parameters (e.g., pacing rate, pacing duration) is likely to affect the profile of metabolic remodeling. Anyway, using (whenever necessary) the protein/wet weight ratio of 0.13 [7], it may be useful to compare our baseline values (obtained from healthy dogs) with those reported previously. Our baseline levels of ATP, ADP, ∑Ad and PCr are comparable to levels reported previously [7,28,29]. However, our baseline ∑Cr level (converting from gram protein to wet weight) is 20 or 80% higher than the levels reported by Shen et al. [7] and Cha et al. [29], respectively. Lastly, our baseline AMP level is 2–4 times higher than previously reported [7,28]. The reason for these deviations is not clear, but may be related to differences in dog breed and/or age. Indeed, our own previous measurements in adult mongrel dogs using exactly the same HPLC setup yielded much lower levels of AMP and ∑Cr (Shibayama, unpublished data), which would be almost identical to values reported by Montgomery et al. [28] and Shen et al. [7], respectively.

Regarding energy metabolism alterations caused by HF, it is interesting to compare our results with the findings of the longitudinal analysis of energy metabolism in a canine pacing-induced DHF model carried out by Shen at al. [7]. These authors reported a progressive reduction of myocardial creatine between 1–2, 3–4, and 7–9 weeks of tachypacing in canine failing hearts [7]. This study also showed that myocardial ATP progressively decreased with the time of pacing, however, the notable reduction was observed only after 3–4 weeks of pacing (11.4% of reduction from basal level in 3–4 weeks, 20.2% in 7–9 weeks). Similar to their 1–2 week stage, our SHF model shows a slight decrease in creatine in the absence of a significant decrease in ATP or adenine nucleotide pool. Likewise, similar to their 3–4 week stage, our DHF model shows a dramatic decrease in creatine concomitant with a moderate decrease in ATP and ∑Ad. These comparisons suggest that our SHF and DHF models represent the relatively early and intermediate stages of metabolic remodeling in HF, respectively.

Shen et al. postulated that the decrease in myocardial creatine is compensatory to the loss of the total adenine nucleotide pool, helping to maintain ATP/ADP ratio at the baseline level [7]. However, in the cited study a significant decrease in creatine preceded decrease in ATP and ∑Ad, and the loss of creatine occurred at a rate 7 times faster than loss of ATP [7]. In our SHF model there was a clear trend towards a decrease in creatine level (p = 0.053) amid otherwise unchanged profile of energy metabolism. Thus, whether the creatine loss is an adaptive response to the ATP loss or a primary event in HF metabolic remodeling remains an open question. Flux through the creatine kinase shuttle, the fastest channel of ATP supply for energy-demanding processes, is determined by the product of the creatine kinase activity and the intracellular creatine concentration. It is of interest that both cytosolic and mitochondrial isoforms of creatine kinase were upregulated in both SHF and DHF models. Moreover, cytosolic creatine kinase was upregulated to a higher level in DHF than in SHF, in inverse proportion to the level of creatine. Thus, in both HF models an increased creatine kinase protein level at least partially compensated for creatine loss. However, previous studies showed that in severe HF the levels of both creatine and creatine kinase activity are reduced [26], suggesting that upregulation of creatine kinases is a transient phenomenon in the course of HF progression.

In our DHF model we found a decrease in ATP without reciprocal increase in ADP and AMP, indicating a loss of the total adenine nucleotide pool (Fig. 3). This, again, is in agreement with findings by Shen at al. [7] who showed a decrease in ATP without changes in ADP and AMP with HF progression. This suggests an imbalance between ATP synthesis and ATP degradation. Whether ATP synthesis was compromised in our DHF model remains unclear. Only a small fraction of subunits comprising Complexes I—V of the electron transport chain were detected. Out of 18 subunits of the Complexes that were detected in our proteomics, 4 were significantly upregulated and 3 were significantly downregulated (see Fig. 2). Notably, the gamma subunit of the ATP synthase catalytic core was upregulated in both SHF and DHF. However, cytochrome b-c1 subunit 2 (gene name UQCRC2) required for assembly of Complex III, was upregulated in SHF but downregulated in DHF. Of interest, two detected subunits of Complex IV were both upregulated in DHF. Overall, the picture is mixed but it does not seem that the electron transport chain was uniformly downregulated in DHF, although no definitive conclusion can be made. In summary, the mechanism of the loss of adenine nucleotide pool in DHF remains unclear and warrants further investigation.

### Glycolysis and glucose metabolism

The “snapshot” data obtained by metabolomics screening does not allow for a direct assessment of substrate flux through the glycolytic pathway. However, an increase in glucose oxidation (in parallel to a decrease in FAO) was consistently demonstrated in canine pacing-induced HF model [10,30]. This “substrate switch” is a hallmark of metabolic remodeling in HF [10,31,32] (reviewed in [33,34]). Assuming that in our study glycolytic flux was also increased, we can make inferences analyzing the profile of changes in individual metabolites and glycolytic enzymes.

We observed a reduction in glycolysis intermediates in both SHF and DHF, without significant changes in myocardial and blood glucose levels. This pattern is consistent with upregulation of glycolytic flux not properly matched by the substrate supply. The fact that the tissue level of glucose was not altered in HF, but all intermediates starting from glucose 6-phosphate were reduced, suggests that the rate-limiting step was the phosphorylation of glucose catalyzed by hexokinase. This assumption is difficult to reconcile with observation that the protein level of hexokinase 1 was highly upregulated in SHF. However, the level of this enzyme was unchanged in DHF, and yet in both models there was a significant decrease in glucose 6-phosphate. The interpretation of the detected hexokinase 1 level may be confounded by the possibility that the distribution of this enzyme between mitochondria and cytosol changed in SHF, without a significant change in the enzyme activity. It is possible that our proteomic analysis detected only cytosolic fraction of the enzyme. It should be noted, however, that degree at which hexokinase 1 can be redistributed between cytosol and mitochondria is controversial [35,36]. In any event, the reduction in the level of glucose 6-phosphate was not in agreement with detected levels of hexokinase 1 protein in this study. Considering other detected glycolytic enzymes, one was downregulated in DHF, and several were upregulated in each of the HF models. Lei et al. found either no change or mild downregulation of key enzymes of the carbohydrate metabolism in severe pacing-induced HF, yet glucose oxidation was 2.5 times higher in failing compared to normal hearts [30]. These authors argued that the increase in glucose oxidation is the consequence of reduced FAO, relieving the inhibition of glycolysis through the mechanism of Randle cycle [21]. Thus, the observed upregulation of some glycolytic enzymes in our study may have a limited role in control of glucose oxidation during HF.

The possibility that glycolysis in HF is limited at the level of hexokinase is consistent with some previous studies suggesting that hexokinase activity becomes more important during states of high glucose fluxes, such as moderate exercise in skeletal muscle [37,38]. It is of interest that in patients with idiopathic dilated cardiomyopathy (DCM) the basal level of cardiac glucose utilization is already as high as the level achieved in normal individuals in response to increased workload due to rapid atrial pacing, and in DCM patients there is no further increase in glucose utilization in response to pacing [39]. Since pacing-induced HF recapitulates many features of DCM phenotype [40,41], our study suggests that glycolytic flux in DCM patients is close to absolute maximum, possibly determined by the maximal hexokinase activity. Under these conditions, lactate could provide a valuable input to the TCA cycle, bypassing the narrow channel imposed by hexokinase. Our data shows severe lactate depletion in pacing-induced HF, suggesting a preferential conversion of lactate to pyruvate with subsequent oxidation in the TCA cycle. Most studies reported unchanged cardiac lactate uptake in pacing-induced HF [10,42]. This is in disagreement with one study showing upregulation of lactate transporter (MCT1) and increased rate of lactate uptake in a rat model of congestive heart failure due to myocardial infarction [43]. The discrepancy may be due to different species or different etiology of the disease. In summary, or study suggests a mismatch between supply and demand in glycolysis and glucose oxidation pathways in pacing-induced HF.

### Fatty acid metabolism

A decrease in FAO has been demonstrated previously in many experimental models of advanced HF and also in patients with severe HF [10,30,39,44]. Thus, this seems to be an undisputable feature of metabolic remodeling in HF. However, it is much less clear how and when in the course of HF the limitation of FAO occurs. Many previous studies showed a decrease in gene and protein expression and activity of key FAO enzymes, such as CPT1 and Medium-chain acyl-CoA dehydrogenase (MCAD), in severe HF [10,30,31,45]. At the same time, FAO remained at the normal level in a moderate severity HF [46]. It is also important to note that downregulation of gene expression of enzymes involved in FAO is often far more pronounced that the changes in the protein levels or enzymatic activity [30,47]. It is possible that downregulation of gene expression occurs early in the course of HF development, however it is confronted by a (yet unknown) mechanism acting at the translational or posttranslational levels to maintain normal FAO [31]. The transcriptional control of genes encoding for FAO enzymes is in large part mediated by peroxisome proliferator-activated receptor (PPAR)-α, the retinoid X receptor-α (RXR-α) and the PPAR co-activator γ (PGC-1α). However, no consistent relationship between the expression of these transcriptional factors and downregulation of FAO has been demonstrated in clinical and experimental HF (reviewed in [34]).

Our study further emphasizes the complexity of FAO control in HF by showing a mixture of downregulation and upregulation in FAO pathway in both models of pacing-induced HF, albeit at a different degree. A unique observation in our SHF model is the significant *increase* in the protein level of CPT1, the enzyme catalyzing the rate-limiting step of FAO. There was a slight decrease in the CPT1 protein level in DHF, but it did not reach statistical significance. Another noteworthy feature of FAO remodeling is a progressive *upregulation* (more in DHF than in SHF) of 3-hydroxyacyl CoA dehydrogenase, the enzyme catalyzing the third step in the cyclic process of FAO.

In addition, the expression level of electron transfer flavoprotein (ETF), which, together with ETF dehydrogenase (ETFDH) transfers high energy electrons from FADH_2_ to the respiratory chain, was significantly increased in DHF, although the expression level of ETFDH was similar between Control and DHF. Among the detected protein related to FAO, only carnitine O-acetyltransferase (CRAT), which plays a role in transferring excess acetyl-CoA from mitochondria to cytosol by forming acetyl-carnitine with carnitine, was significantly decreased in DHF (but not in SHF).

Thus, it is clear that in our study there was no significant downregulation of FAO enzymes, which would be consistent with findings in a microembolization-based model of moderate HF in the dog [46]. However, it is important to note that the limitation of FAO flux in our study could have occurred due to the decreased myocardial level of carnitine. Carnitine is required for free fatty acid uptake in mitochondria for β-oxidation and is the substrate of CPT1, forming acylcarnitine species from carnitine and fatty acyl-CoA. Clearly, limited availability of carnitine can downregulate the FAO flux in a way similar to a decreased activity of CPT1. Myocardial free carnitine level was significantly reduced in DHF and slightly, but not significantly, reduced in SHF. Of interest, despite large differences in the levels of free tissue carnitine and CPT1 between SHF and DHF, the majority of acylcarnitine species were significantly (and to a similar degree) decreased in both SHF and DHF (see Fig. 7). This could suggest that in both models FAO flux is limited by the availability of CPT1 substrates (carnitine, and possibly free fatty acids in DHF) rather than by CPT1 activity.

It is of interest that the plasma levels of carnitine and some acylcarnitines were significantly elevated in post-pacing DHF, suggesting that carnitine uptake and/or removal in DHF was modified. Carnitine uptake is mediated by a sarcolemmal Na^+^-dependent transporter, organic cation transporter novel type 2 (OCTN2). OCTN2 concentrates carnitine inside cells using energy stored in the transmembrane sodium gradient. The intracellular Na^+^ concentration was shown to be elevated (and thus the transmembrane Na^+^ gradient reduced) in some HF models [48]. In addition, the protein expression of Na^+^/K^+^ pump β-subunit was significantly downregulated in our DHF model (see S9 Fig.). Thus, at least in part the reduced carnitine uptake in DHF could be due to reduced transmembrane Na^+^ gradient. A recent study showed that the expression of OCTN2 decreased in dilated cardiomyopathy in human patients and mice [49]. Also, elevated plasma levels of carnitine and some acylcarnitines have been reported in patients with HF, and plasma carnitine level correlated significantly with impaired left ventricular systolic function [50]. Moreover, the plasma levels of some acylcarnitine species significantly correlated with the New York Heart Association functional class, further supporting the link between the disturbed carnitine metabolism and the severity of HF [51]. However, the molecular mechanisms responsible for increasing plasma carnitine pool in HF remain largely unknown. It seems unlikely that reduced uptake by the heart muscle alone would be sufficient to increase the plasma concentration by more than 60%. We speculate that skeletal muscle might undergo parallel remodeling of carnitine metabolism in HF. In support of this idea, Marin-Garcia et al. demonstrated similar remodeling of oxidative phosphorylation occurring simultaneously in cardiac and skeletal muscle in pacing-induced failing hearts, presumably mediated by the tumor necrosis factor (TNF)-α signaling [52]. However, the extent of fatty acid metabolism remodeling in skeletal muscle has not been studied in pacing-induced HF.

Summarizing the above, the major difference between SHF and DHF is in the protein level of CPT1 (significantly increased only in SHF) and the tissue level of carnitine and some fatty acids (significantly reduced only in DHF). It is tempting to assume that SHF represents an earlier (and hence more moderate) stage of DHF, at which metabolism is largely compensated. However, given the lack of significant downregulation of FAO enzymes in DHF, this model probably also represents a moderate degree of the disease (which would be consistent with moderate degree of energy decompensation and diastolic failure as compared to previous reports using DHF models, as discussed above). If this is true, then the decrease in the cellular acylcarnitine pool may be a primary mechanism limiting FAO and reducing energy stores early in HF progression. To prove this point, a serial study of different time points in the same model of HF will be required.

### TCA Cycle

We are not aware of any prior study comparing TCA cycle intermediates and the expression of its enzymes in the same model of HF. Most of the previous relevant studies showed *decreased* protein levels of some TCA cycle enzymes in various HF contexts (in human dilated cardiomyopathy [53]; in pacing-induced rabbit HF model [54]; in rat post-infarction HF model [55]). Also, a decrease in mRNA level of some TCA cycle enzymes was previously reported in a pacing-induced canine HF model very similar to our DHF model [9]; however, judging from the publically available gene chip data (Gene Expression Omnibus accession No. 9794), the gene expression of most of the TCA cycle enzymes was essentially unchanged throughout the entire period of that longitudinal HF study (3 days, 1 week, 2 weeks, and end-stage pacing-induced HF [9]).

In contrast to those studies and in accord with ours, Bugger et al. reported an increase in protein levels of two out of six TCA cycle enzymes detected, mitochondrial aconitase and succinate dehydrogenase complex, subunit A, in a rat model of aortic constriction-induced HF [19]. Other detected TCA cycle enzymes were unchanged in that study. It is of interest that we found an increase in the same two enzymes (in addition to a dramatic upregulation of citrate synthase) in our SHF model. However, in our DHF model the pattern of upregulation shifted, now showing significantly increased levels of 2-oxoglutarate dehydrogenase, fumarase, and malate dehydrogenase (see Fig. 5). In any event, no downregulation of the TCA cycle protein expression occurred in our SHF and DHF models.

The unique, and unprecedented finding of our study is the opposite direction of change in the levels of intermediates versus enzymes of the TCA cycle in our HF models. Four detected intermediates (2-oxoglutarate, succinate, fumarate, and malate) were decreased in SHF, and, in a seemingly backward progression, the 3 intermediates of the first half of the TCA cycle (citrate, aconitate, isocitrate) joined the pool of deficient intermediates in DHF. Overall, it appears that the TCA cycle was strained for substrate inputs (i.e., experienced anaplerotic deficit). Whether the upregulation of the TCA cycle enzymes was a cause or a consequence of the substrate depletion is uncertain. Our preferred speculation is that the upregulation of the TCA cycle enzymes is a direct compensatory response to the increased mechanical stress associated with rapid pacing, whereas the depletion of intermediates is secondary and just reflects of the mismatch between the supply and demand. In this case the more severe depletion of TCA cycle intermediates in the DHF model would be consistent with the higher level of stress due to dyssynchronous contraction.

An interesting question is why anaplerosis, the physiological process of replenishing the pool of the TCA cycle intermediates [20], did not compensate for the TCA cycle depletion. Pyruvate carboxylation to oxaloacetate or malate is considered to be a major anaplerotic pathway [18]. Additional pathways include (1) transamination reactions between oxaloacetate and α-ketoglutarate and their corresponding amino acids, aspartate and glutamate, and (2) formation of succinyl-CoA from propionyl-CoA precursors such as branched-chain amino acids, propionate, and odd-carbon ketone bodies and fatty acids [18]. It is plausible that the anaplerotic pathway through pyruvate carboxylation pathway was limited simply by the availability of pyruvate. Recall that pyruvate was reduced in both SHF and DHF (see Fig. 8). Although the reduction in tissue pyruvate was statistically significant only in SHF, the trend of the overall decrease in the levels of all glycolytic intermediates, including pyruvate, was clearly visible in DHF as well. As we argue in the S1 Text, it is unlikely that this trend, consistent among all detected intermediates, is due to random measurement error. Also, a severe reduction of the tissue lactate (a significant source of pyruvate under normal aerobic conditions) in both SHF and DHF further supports the assumption that the TCA cycle was “pyruvate-starved” in both models of HF. Regarding the anapletoric pathways involving amino acids, we found no decreases in these potential sources of the TCA cycle replenishment. In fact, three branched-chain amino acids (valine; leucine; isoleucine) were increased in both SHF and DHF, although only leucine in SHF was significantly different from Control. Other relevant amino acids (i.e. glutamate, glutamine, aspartate) did not show any significant changes either in SHF or DHF. Thus, it appears that the anaplerotic pathways associated with amino acids were not able to compensate for the deficit in the TCA cycle intermediates in our HF models, and this was not due to a decrease in the levels of participating amino acids. This supports the notion of the critical role of pyruvate as the main source of anaplerosis in the heart [18].

### Comparison with previous studies using pacing-induced HF models

Our SHF and DHF models both showed a prominent and similar degree of systolic dysfunction (LV developed pressure decreased by ~30% and ~40% in SHF and DHF, respectively). In contrast, diastolic dysfunction was not present in SHF and was very moderate in DHF (see Table 1). In previous studies using very similar pacing protocols in the dog the degree of systolic dysfunction was comparable to that in our study (LVESP was reduced by ~20% and ~35% in SHF and DHF, respectfully [6]), but the level of diastolic dysfunction was much larger than in our study, and was not different between SHF and DHF [6,8]. This was a somewhat unexpected outcome. The most likely reason is the difference in age and/or breeding conditions of animals. In particular, it is likely that dogs used in our study were younger than those used in previous studies of pacing-induced HF. Other factors complicating comparison of results from different studies include differences in pacing rate and the location of the pacing electrode, and whether the hemodynamic measurements were performed in conscious or anesthetized animals.

One of early studies using canine model of pacing-induced HF revealed that systolic dysfunction develops early (after 1 week of pacing in that study), and does not increase much with progression of the disease towards the end stage, whereas diastolic dysfunction develops later [40]. This is concordant with another study, in which an analysis of energy metabolism was performed at different stages (1–2 weeks, 3–4 weeks, and 7–9 weeks) of pacing-induced HF in a canine model [7]. In that latter study the maximum decrease in LVSP (by ~20%) had already occurred at 1–2 weeks of pacing, whereas LVEDP continued to increase and reached plateau only after 3–4 weeks of pacing [7]. It should be noted however that the above mentioned two studies [7,40] used a higher rate of pacing (240 bpm vs. 200 bpm in our study), which is expected to speed up progression of HF. Comparing metabolic outcomes, in the above mentioned study by Shen et al. [7], the earliest feature of metabolic remodeling (after 1–2 weeks of pacing) was a 15% decrease in tissue creatine, whereas the ATP level remained normal. After 3–4 weeks of pacing, however, creatine decreased by 28% and ATP decreased by 12%. In our SHF model creatine decreased by 11% with no decrease in ATP, and in our DHF model creatine decreased by 35% and ATP decreased by 13%. Based on the above, it is reasonable to position the outcomes of our SHF and DHF models as representative of an early and intermediate stage of HF, respectively. The principal difference between SHF and DHF is that the overt energy deficit is present only in DHF. As discussed above, this may be related to a significant decrease in the tissue levels of carnitine and some long-chain fatty acids (limiting the substrate flux through FAO) in DHF, but not in SHF. Why and how the presence of electromechanical dyssynchrony (other things being equal) is translated into the decreased tissue levels of carnitine and long-chain fatty acids is a mystery. However, it seems to be unlikely that these metabolic alterations are mediated via regulatory circuits local to the heart. The reciprocal changes in the plasma carnitine (see S3 Fig.) suggest involvement of organism-level control mechanisms, which might be more activated in DHF due to a larger level of cardiac stress. Whether indeed SHF represents the earlier phase of DHF, or the two models follow different trajectories of metabolic remodeling, remains uncertain. Answering this question will require the analysis of earlier and later time points during the progression of the SHF and DHF models.

### The state of conflict

Assuming that our SHF and DHF models represent the early and intermediate stages of HF, our findings suggest that the metabolic system passes through a “state of conflict” during progression of the disease. In the most general terms, this is a conflict between limitation of substrate availability and upregulation of many enzymes involved in the FAO, glycolysis, the TCA cycle, and the mitochondrial respiratory chain. This general description principally applies to both SHF and DHF, although there are differences in many specific features between the two HF models.

We are not aware of any prior work showing such discordance between the changes in levels of metabolites and metabolic enzymes in the course of HF. There are several possible reasons that this pattern remained undetected. Relatively few studies addressed metabolic changes at intermediate stages of HF and still fewer (if any) combined extensive metabolomics and proteomics in one study, which was necessary to reveal the conflict. In fact, many specific observations of or study are in line with previous reports focused on particular metabolic pathways/subsystems or using a subset of techniques used in our study. For example, Bugger et al. noted upregulation of some TCA cycle enzymes, as well as some subunits of the electron transport chain Complexes even at a presumably more advanced stage of HF than in both our HF models [19]. Williams et al. observed upregulation of creatine kinase (B type) at the mRNA level in their canine pacing-induced HF model presumably producing a moderate HF phenotype. Yet, in a different study Shen et al.[7] identified the decrease in tissue creatine as the earliest metabolic manifestation of the disease in a canine DHF model similar to that used by Williams et al. [40] as well as our DHF model. The combination of the latter two findings is in full agreement with our data; we just showed that they co-exist in the same hearts, highlighting the discordant pattern of the creatine kinase system remodeling in pacing-induced HF.

Identification of the conflicting pattern in metabolic remodeling offers important insights into the pathogenesis of HF. The “energy starvation” hypothesis was proposed as a paradigm of metabolic decompensation in HF [56–58]. Two widely accepted causes of energy starvation are (1) mitochondrial damage [19,52,59,60] and/or (2) coordinated downregulation of energy metabolism (possibly by reduced transcription of genes regulated by PPAR-α/RXR-α/PGC-1α heterotrimer or PPAR-α-Sirt1 complex [10,32,60–64] and possibly affecting genes coding for enzymes involved in FAO, the TCA cycle and mitochondrial oxidative phosphorylation [9,65]. However, the role of these two phenomena and the stage of HF at which they are operative remains a matter of a considerable debate [47,66]. For example, a recent study showed that mitochondria isolated from hearts of patients with end-stage HF are able to maintain normal respiratory function [66]. Also, the regulation and the dynamics of gene and protein expression during progression of HF is a highly complicated matter, as reviewed recently by Lionetti et al. [34]. For one thing, the changes in the mRNA and protein levels of metabolic enzymes during HF are not always identical. In some cases the reduction in mRNA is much more pronounced (or is ahead of) the reduction in the protein content, and even the direction of change in gene and protein expression may not coincide [19,31]. Even more importantly, the profiles of gene and protein expression in human HF patients seem to depend on the etiology of the disease. There is evidence that gene and protein expression of PGC-1α, PPAR-α and at least some of their target genes encoding for enzymes involved in fatty acid metabolism are upregulated in human DCM, but not in HF with other etiologies [67,68]. As it is generally believed that pacing-induced HF model recapitulates many features of the DCM phenotype [40,41], our finding of increased protein expression of many catabolic enzymes is not inconsistent with the clinical studies mentioned above. Unfortunately, the energetic state of the heart was not assessed in those studies. However, our results suggest that energy deficit in intermediate stages of HF may be associated with substrate limitations *despite* upregulation of metabolic enzymes.

The substrate limitations appear to occur simultaneously at many input points of the myocyte metabolism. This includes intracellular carnitine, creatine, lactate, and some long-chain fatty acids. The glucose uptake is not limited, but the limitation of glycolysis occurs at the level of glucose 6-phosphate, leading to a decrease in the levels of all downstream glycolytic intermediates. As discussed above, glycolytic flux in HF may be close to saturation and limited by the maximal activity of hexokinase. The upregulation of catabolic enzymes may be an adaptive response to substrate limitations. Whereas the decrease in glycolytic intermediates and long-chain FAs can be explained simply by a mismatch between supply and demand, the decrease in cellular uptake of carnitine and creatine looks more like a part of a deliberate programmatic downregulation. It is difficult to accept the restriction in availability of these important carrier molecules as adaptive, although the decrease in creatine may be important to maintain ATP/ADP ratio amid decreasing level of ATP [26]. It is of interest that the decrease in myocardial levels of both carnitine and creatine appears to be a universal feature of metabolic remodeling in both experimental and human HF. Individually, the decrease in each of these carrier molecules was shown to correlate with the severity of the disease in HF patients [27,49]. Our study shows that the downregulation of these two molecules might be coordinated, judging from parallel reduction of carnitine and creatine in SHF and DHF models (compare Figs. 3C and 7A). Surprisingly, regulatory mechanisms mediating the decreased uptake of carnitine and creatine in HF remain unknown.

Finally, it should be emphasized that “the state of the conflict” discussed above is probably a transient phenomenon in the evolution of HF. Indeed, one can see that in DHF (presumably representing a later stage of HF than SHF) fewer detected metabolic enzymes are upregulated, more are downregulated, and some enzymes are reduced in DHF as compared to SHF. In particular, the protein level of CPT1 is upregulated in SHF, but in DHF it is trending lower than in Control. Thus it is plausible that with longer time of pacing in the DHF model we would found a metabolic profile in which both metabolites and metabolic enzymes are uniformly reduced. On enzymatic level, this would reconcile our findings with those obtained in studies analyzing more advanced stages of canine pacing-induced HF [10].

## Conclusion

Our integrative metabolomics and proteomics study shows that the presence of electromechanical dyssynchrony in moderate pacing-induced HF significantly affects the profile of metabolic remodeling, the principal difference being the presence of energy deficit in DHF, but not in SHF. At least in part, the reduced energy reserve in DHF is due to a much more pronounced decrease in tissue levels of carnitine and creatine than in SHF. However, the regulatory pathways linking mechanical abnormality with a more adverse metabolic outcome remain elusive. A novel finding in both SHF and DHF models is the apparent conflict between a decrease in tissue levels of many metabolic substrates/intermediates coincident with upregulation of many catabolic enzymes. This pattern suggests substrate limitation as an early metabolic abnormality in the course of pacing-induced HF, possibly preceding systemic downregulation of oxidative metabolism and/or mitochondrial damage. Future studies should apply combined—omics approaches at different time points in HF progression in order to fully decipher the intricate relationship between adaptive and maladaptive metabolic remodeling in the course of HF.

### Limitations

Our ability to collect biopsies from the failing hearts were limited due to the fact that our study was a satellite to a different project, which used the failing hearts for *ex-vivo* studies. We could introduce only a minimal delay in the main protocol of that project, and only a minimal damage to the hearts. Therefore, a limited amount of cardiac tissue (~100 mg) was collected, and only from the apical anterior LV epicardium. Regional heterogeneity in DHF has been reported, including gene and protein expression [8,65], calcium handing and subcellular structural remodeling [3,5] and electrophysiological remodeling [5,69]. Due to late contraction in the LV lateral wall it undergoes greater mechanical stress; thus it is conceivable that the metabolic remodeling is also more exaggerated in that region. However, our metabolomic analysis demonstrated that significant metabolomic alterations occurred in DHF as compared to SHF and normal hearts, even in the region of LV not undergoing the largest mechanical stress. Moreover, the elevated plasma carnitine and acylcarnitines indicated that a systemic modification of metabolism occurred in dyssynchronous failing hearts. The constraints indicated above also limited our ability to allot tissue for additional analyses, such as gene expression and assessment of enzymatic activity of key proteins. Finally, the proteomic analysis of the whole tissue extracts was unable to detect membrane-bound proteins responsible for uptake of substrates and carrier molecules (carnitine and creatine). This limitation warrants additional analyses of the membrane-enriched fraction, which was not possible in current study due to limited amount of collected tissue.

## Supporting Information

S1 Dataset The complete set of myocardial metabolome. (XLSX)

S2 Dataset The complete set of plasma metabolome. (XLSX)

S3 Dataset The complete set of detected proteins. This includes all the proteins that were detected in all 3 technical replicates of all analyzed samples.
(XLSX)

S4 Dataset Correlation analysis of glycolytic intermediates. See S1 Text for details.
(XLSX)

S1 Fig Ratio of ATP over the total level of ADP and free ADP (ADP_f_). ADP_f_ was computed based on the assumption of equilibrium between ADP and creatine via creatine kinase reaction [7]. *p<0.05
(TIF)

S2 Fig Changes in plasma levels of carnitine and acylcarnitines in SHF and DHF after 6 weeks of pacing. Values obtained after pacing (“post”) are presented as the percent of the values obtained in the same animals before pacing (“pre”). *p<0.05 by paired *t*-test.
(TIF)

S3 Fig Total pools of free and conjugated carnitine in myocardium and plasma. The sum of carnitine and all acylcarnitines was calculated in myocardial (**A**) and plasma (**B**) samples from Control, SHF, and DHF animals. (The levels of individual acylcarnitines can be found in Fig. 7 (myocardium) and S7 Fig. (plasma)). Note that the total pool of myocardial carnitine significantly *decreased* while the total pool of plasma carnitine significantly *increased* in DHF as compared to Control. *p<0.05
(TIF)

S4 Fig Individual data points for glucose and 9 glycolytic intermediates measured by GS/MS. See S1 Text for discussion of these data. DHAP: dihydroxyacetone phosphate.
(TIF)

S5 Fig Comparison of maximal values of 9 glycolytic intermediates between the Control, SHF, and DHF groups. The maximal values of each intermediate were normalized to control and averaged among intermediates. The graph shows that the maximal values were consistently smaller in both SHF and DHF compared to control. Since the set of tested intermediates includes molecules with different mass and charge, it is unlikely that the consistent decrease in the maximal values in the HF groups is due to the measurement error. It would be consistent, however, with the assumption that the tested intermediates oscillate in the range from close to zero to levels close to the maximum levels detected by GS/MS. In this case, the observed decreases in maximal values in the HF models would indicate the decrease in the amplitude of oscillations. *, p<0.0001
(EPS)

S6 Fig Myocardial lactate levels in Control and DHF measured by GC/MS and an enzymatic assay. Consistent to the results from GC/MS analysis (**A**, also shown in Fig. 1), the quantitative measurement using biochemical assay (**B**) showed a significant reduction in the level of lactate in DHF as compared to Control. *p<0.05 (t-test)
(TIF)

S7 Fig Heat map of plasma metabolome. The data obtained by two metabolomic platforms (GC/MS and MS/MS) and presented as fold change in SHF and DHF animals after 6 weeks of pacing (“post”) as compared to those from the animals before pacing (“pre”). Green indicates a significant decrease, and read indicates a significant increase as compared to Control. The ID # in this heat map corresponds to that in the myocardial metabolome heat map (Fig. 1). Note that the most robust and consistent differences between *pre- and post-paced animals in DHF* are found in the plasma levels of carnitine and acylcarnitines, which contrasts to the significant reduction in myocardial carnitine and acylcarnitines (see Figs. 1 and 7). This suggests the global alteration of carnitine metabolism is a prominent feature of organism-level metabolic remodeling in DHF animals. The plasma level of sorbitol remarkably increased after pacing in DHF animals. However, whether this increase is involved in pathophysiology of DHF needs to be elucidated. *p<0.05 (paired *t*-test). BCAA: branched-chain amino acid, GSH: glutathione, GC/MS: gas-chromatography/mass-spectrometry, MS/MS: tandem mass-spectrometry.
(TIF)

S8 Fig 2-aminoadipic acid (2-AAA) as a myocardial marker of heart failure. Myocardial levels of 2-AAA (**A**) and blood glucose levels (**B**) in Control, SHF (post-pacing) and DHF (post-pacing). The myocardial levels of 2-AAA, previously shown as a plasma biomarker of diabetes [70], were significantly decreased both in SHF and DHF hearts as compared to Control. However, the blood glucose levels were very similar in all three groups, indicating that SHF and DHF animals did not develop hyperglycemia. The reduction in myocardial 2-AAA level was more pronounced in DHF than SHF.
(EPS)

S9 Fig Protein expression of Na^+^/K^+^ pump subunits in Control, SHF, and DHF. Protein levels of α-subunit (**A**) and ß-subunit (**B**) of Na^+^/K^+^ pump. The protein level of ß-subunit was significantly lower in DHF than in Control and SHF.
(TIF)

S1 Table Additional statistical analysis of glycolytic intermediates. See S1 Text for details.
(XLSX)

S1 Text Additional results and discussion. This text includes the additional analysis and discussion of glycolytic intermediates, the myocardial content of 2-aminoadipic acid, and the protein expression of Na^+^/K^+^ pump.
(DOCX)
