# Supplementary figures and images for: Metabolic Remodeling in Moderate Synchronous versus Dyssynchronous Pacing-Induced Heart Failure: Integrated Metabolomics and Proteomics Study
Source: PLoS One. 2015 Mar 19;10(3):e0118974. doi: 10.1371/journal.pone.0118974 (PMC4366225; doi:10.1371/journal.pone.0118974)

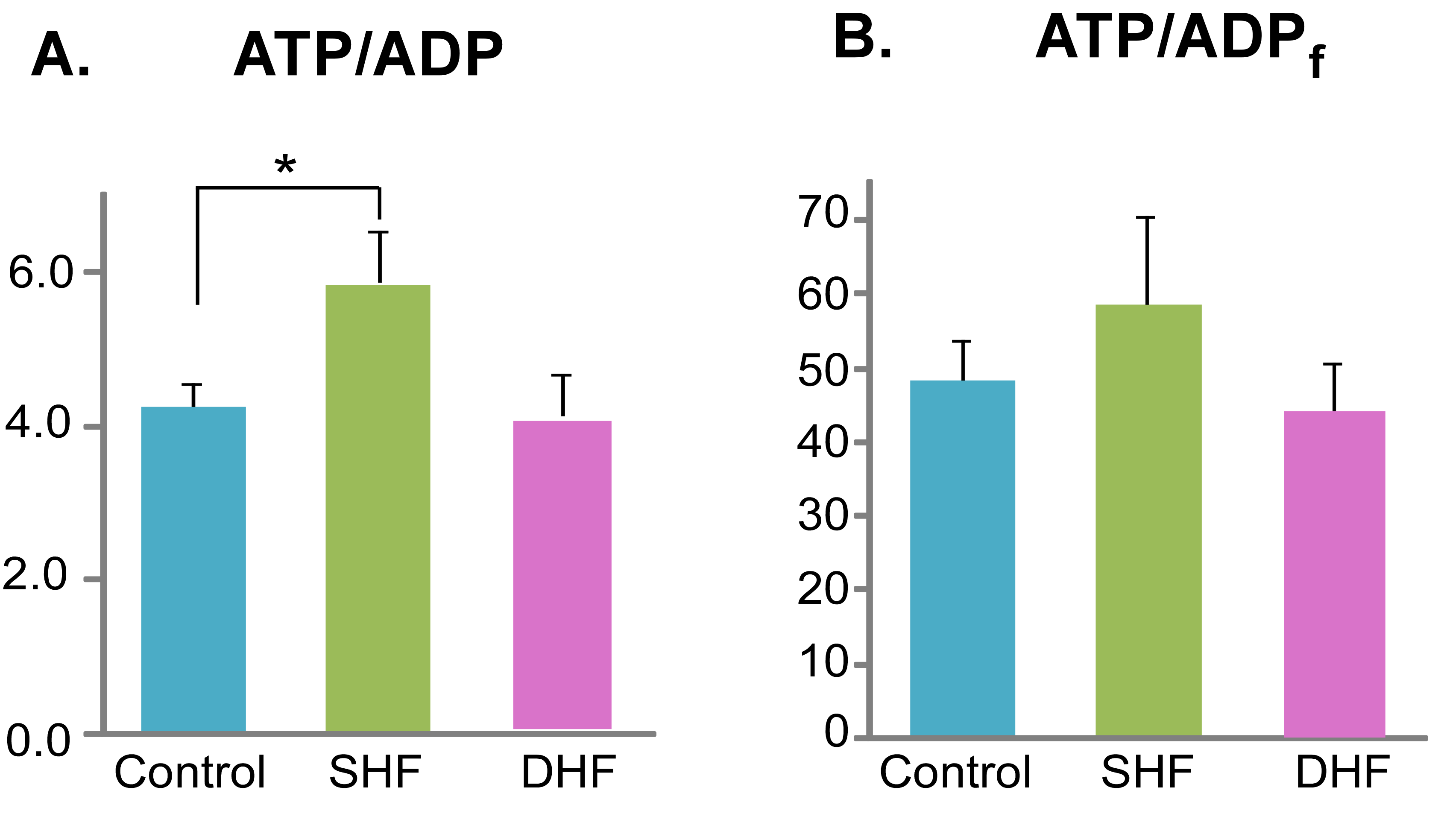

Supplement: S1 Fig — ADPf was computed based on the assumption of equilibrium between ADP and creatine via creatine kinase reaction [7]. *p<0.05 (TIF) [file pone.0118974.s005.tif]

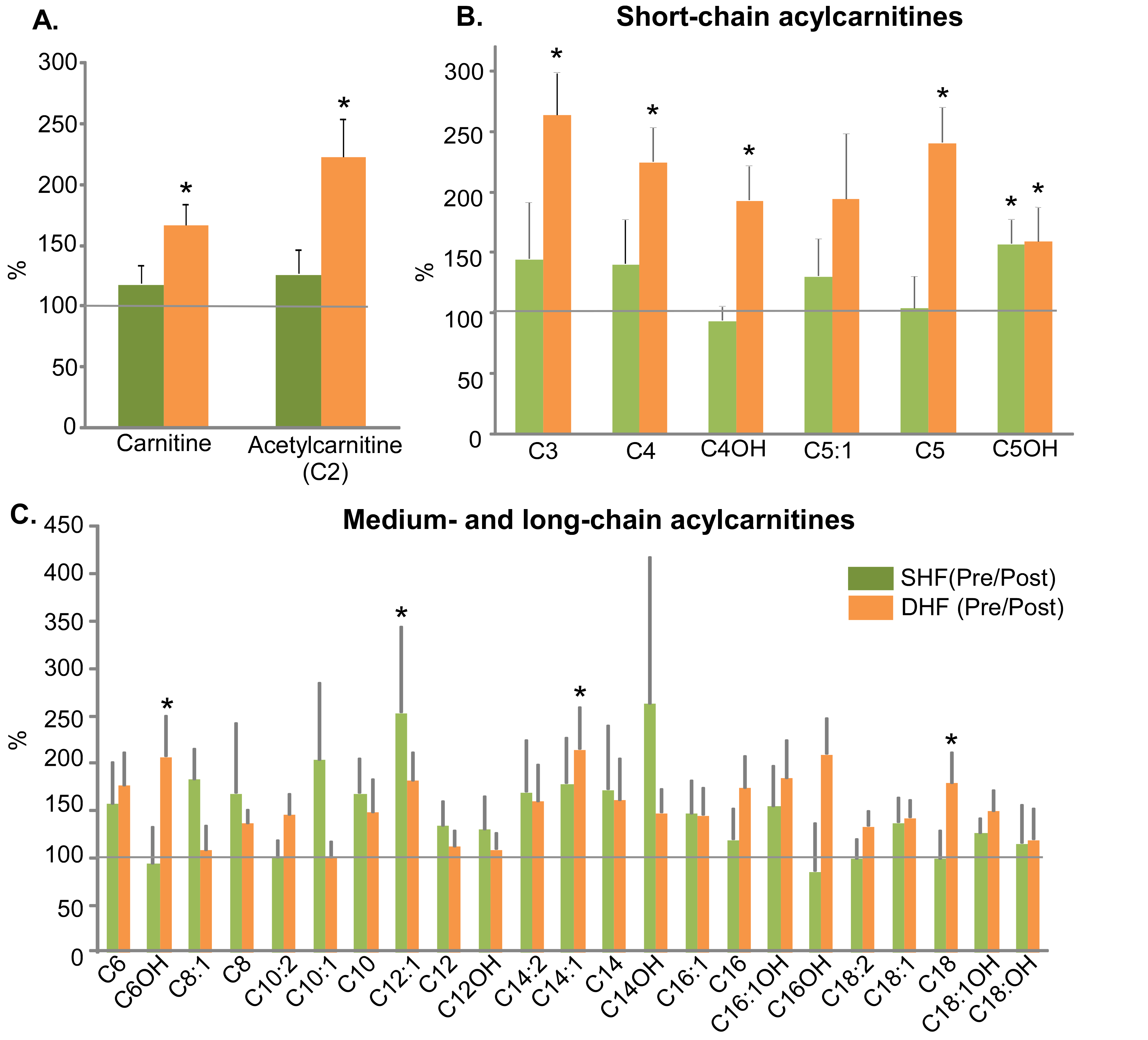

Supplement: S2 Fig — Values obtained after pacing (“post”) are presented as the percent of the values obtained in the same animals before pacing (“pre”). *p<0.05 by paired t-test. (TIF) [file pone.0118974.s006.tif]

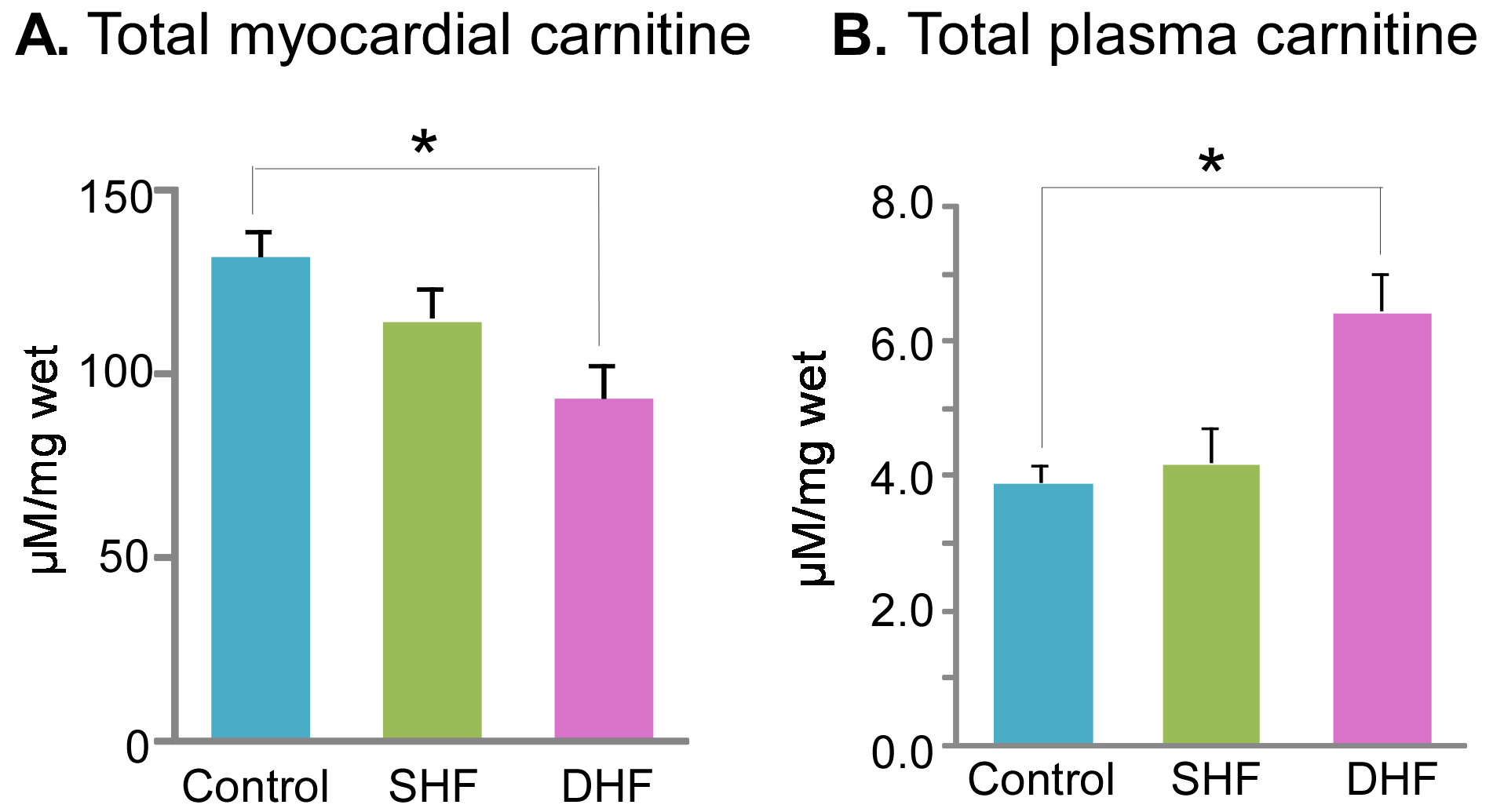

Supplement: S3 Fig — The sum of carnitine and all acylcarnitines was calculated in myocardial (A) and plasma (B) samples from Control, SHF, and DHF animals. (The levels of individual acylcarnitines can be found in Fig. 7 (myocardium) and S7 Fig. (plasma)). Note that the total pool of myocardial carnitine significantly decreased while the total pool of plasma carnitine significantly increased in DHF as compared to Control. *p<0.05 (TIF) [file pone.0118974.s007.tif]

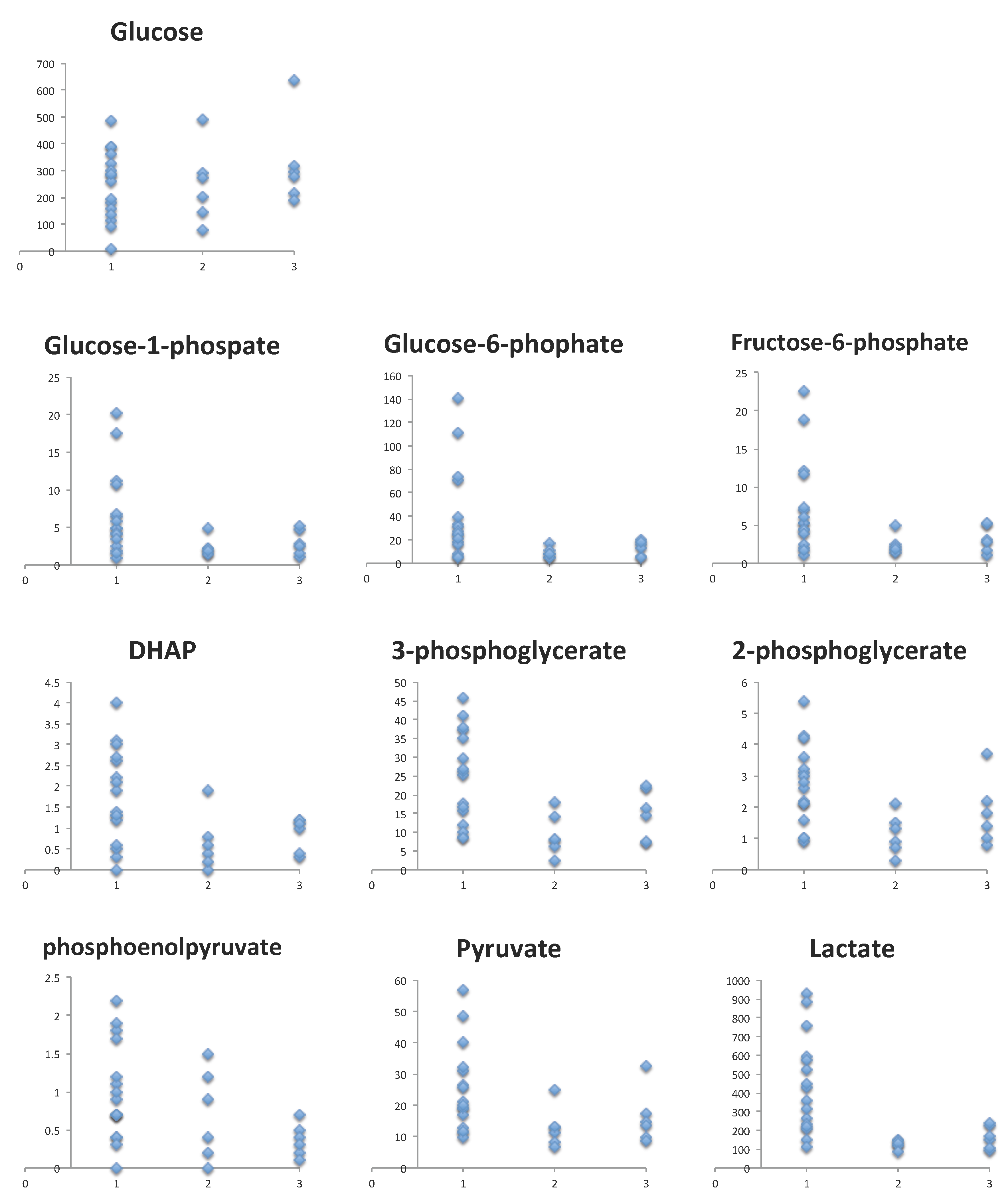

Supplement: S4 Fig — See S1 Text for discussion of these data. DHAP: dihydroxyacetone phosphate. (TIF) [file pone.0118974.s008.tif]

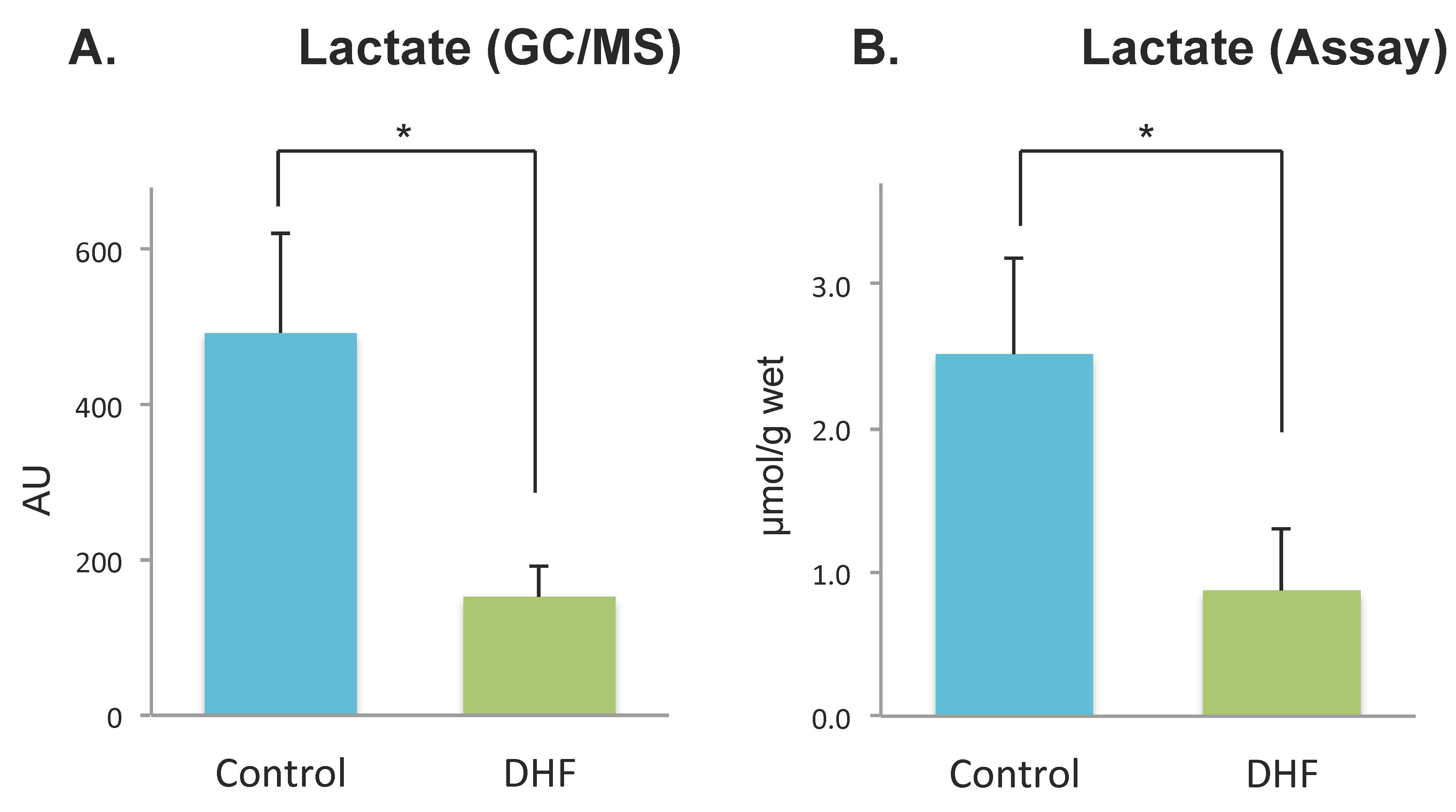

Supplement: S6 Fig — Consistent to the results from GC/MS analysis (A, also shown in Fig. 1), the quantitative measurement using biochemical assay (B) showed a significant reduction in the level of lactate in DHF as compared to Control. *p<0.05 (t-test) (TIF) [file pone.0118974.s010.tif]

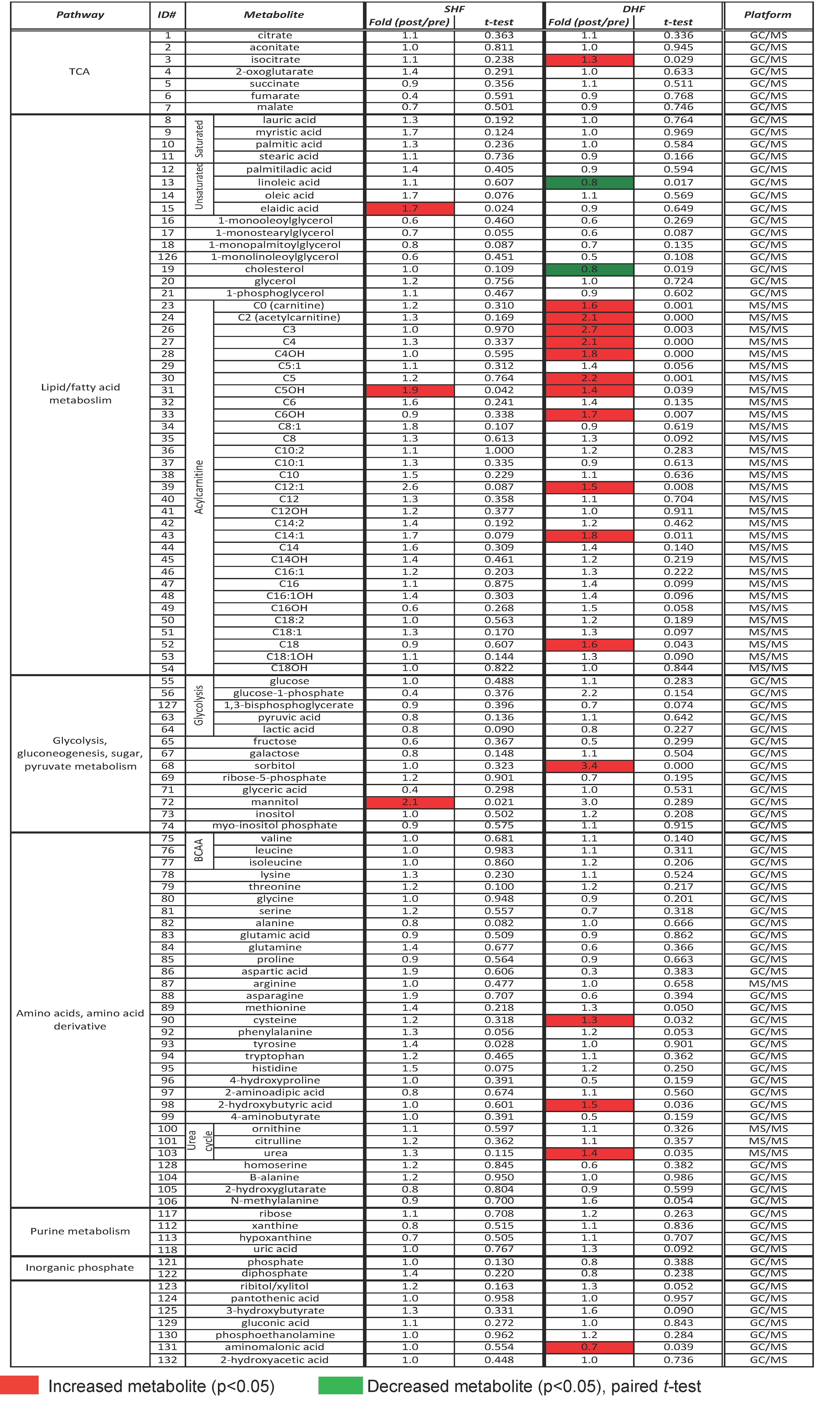

Supplement: S7 Fig — The data obtained by two metabolomic platforms (GC/MS and MS/MS) and presented as fold change in SHF and DHF animals after 6 weeks of pacing (“post”) as compared to those from the animals before pacing (“pre”). Green indicates a significant decrease, and read indicates a significant increase as compared to Control. The ID # in this heat map corresponds to that in the myocardial metabolome heat map (Fig. 1). Note that the most robust and consistent differences between pre- and post-paced animals in DHF are found in the plasma levels of carnitine and acylcarnitines, which contrasts to the significant reduction in myocardial carnitine and acylcarnitines (see Figs. 1 and 7). This suggests the global alteration of carnitine metabolism is a prominent feature of organism-level metabolic remodeling in DHF animals. The plasma level of sorbitol remarkably increased after pacing in DHF animals. However, whether this increase is involved in pathophysiology of DHF needs to be elucidated. *p<0.05 (paired t-test). BCAA: branched-chain amino acid, GSH: glutathione, GC/MS: gas-chromatography/mass-spectrometry, MS/MS: tandem mass-spectrometry. (TIF) [file pone.0118974.s011.tif]

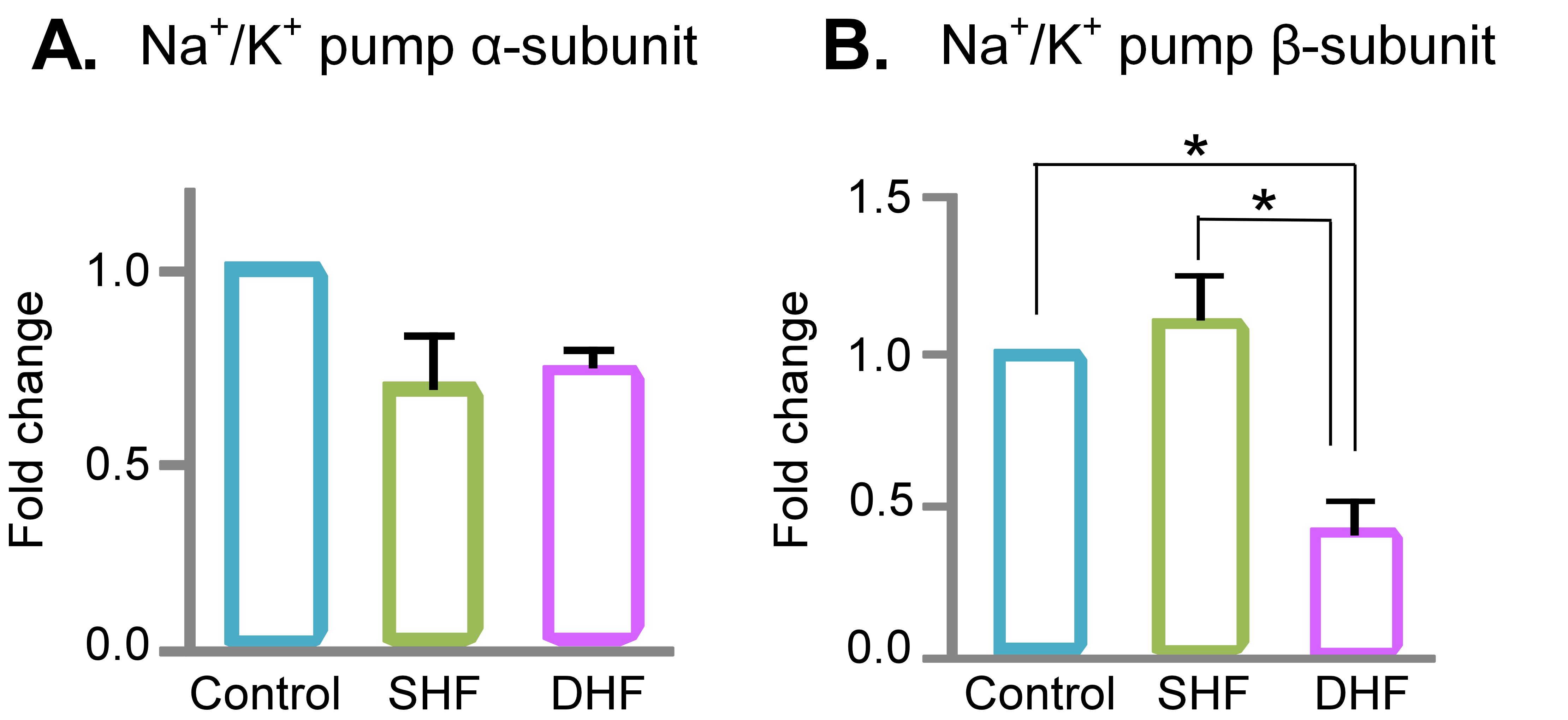

Supplement: S9 Fig — Protein levels of α-subunit (A) and ß-subunit (B) of Na+/K+ pump. The protein level of ß-subunit was significantly lower in DHF than in Control and SHF. (TIF) [file pone.0118974.s013.tif]
