# Supplementary material for: Metabolic Remodeling in Moderate Synchronous versus Dyssynchronous Pacing-Induced Heart Failure: Integrated Metabolomics and Proteomics Study
Source: PLoS One. 2015 Mar 19;10(3):e0118974. doi: 10.1371/journal.pone.0118974 (PMC4366225; doi:10.1371/journal.pone.0118974)
Supplement: S1 Text — This text includes the additional analysis and discussion of glycolytic intermediates, the myocardial content of 2-aminoadipic acid, and the protein expression of Na+/K+ pump. (DOCX) [file pone.0118974.s015.docx]

**Results and Discussion (S1 Text)**

**Metabolic profile of glycolysis – extended analysis**

Individual measurements for tissue glucose and 9 intermediates of glycolysis detected by GC/MS are shown in S4 Fig. What draws attention is that many intermediates (especially G1-P, G6-P, DHAP, phosphoenolpyruvate) have a range of values with the lower bound close to zero in all experimental groups (Control, SHD, DHF). Then, the maximal value of all 9 intermediates is larger in Control than in both HF models. In fact, if we normalize the maximal values of all intermediates in all groups to the respective Control values and compare the average normalized maximal value between the groups, both SHF and DHF groups are significantly different from Control (p<0.0001 for both comparisons, see S5 Fig). So, it seems that the difference in *the ranges* of the metabolites levels between the groups are not due to random error of measurement. This inference is further supported by the fact that many of the intermediates which are linked by one enzymatic reaction correlate between different hearts in the same group. The correlation analysis is presented in the Dataset 4. Note a high degree of correlation (R^2^ > 0.85) between G1-P and G6-P, and between G6-P and F6-P in all groups. Note also that the apparent outliers (very high values of G1-P, G6-P and F6-P observed in the Control and SHF groups, apparently detached from the main cluster of points) do not deviate from the correlation, i.e., occur simultaneously in one or two “neighboring” intermediates. Of interest, even intermediates far apart in the glycolytic chain may correlate (e.g., pyruvate vs. G6-P in the Control group). Assuming that GS/MS detections of different glycolytic intermediates are independent, these observations support the idea that the high or maximal values of at least some glycolytic intermediates are not due to random noise.

We believe that the observed distributions in the values of glycolytic intermediates reflect glycolytic oscillations, which were previously demonstrated in different systems [1,2]. These oscillations are due to various feedback mechanisms in the regulation of glycolytic enzymes by their products and other metabolites [2]. Thus, the range of the observed values does not reflect the measurement noise, but rather the magnitude of fluctuations in the concentrations of analytes. The sampling in different hearts occurs at random phases of the fluctuations, and a sufficient number of random samples should cover reasonably well the full range of the cyclic process.

Postulating the existence of this fundamental phenomenon in the canine ventricular myocardium, we can make several inferences relevant to metabolic remodeling in HF as well as to the GS/MS method. (1) We infer from the data that the amplitude of the oscillations is decreased in both SHF and DHF as compared to control. Of note, the decrease in the amplitude of oscillations occur downstream of hexokinase. The ranges of glucose levels are not decreased in SHF and DHF; if anything, the whole distribution of glucose values might be trending upwards in DHF (see S4 Fig). (2) Most of the intermediates, especially those in the upper part of glycolytic pathway fluctuate “deeply”, reaching levels close to zero. (3) The phase of fluctuations is likely to be distributed along the glycolytic chain, such that some intermediates oscillate in phase, whereas some intermediates oscillate out of phase. This might explain large difference in the coefficient of correlation between different pairs of metabolites. High correlation might reflect oscillation in phase, whereas low correlation might indicate oscillation out of phase. (4) The difference in the correlation relationships along the glycolytic chain between the Control and HF groups (see Dataset 4) might reflect the differences in the dynamic control of the glycolytic pathway. Of particular interest is the fact that in the Control group pyruvate is highly correlated with both G6-P and lactate, whereas in SHF and DHF this is no longer the case. This suggests that the control over pyruvate concentration shifts from glycolysis to other pathways.

On the technical level, (1) the data suggest a relatively high accuracy of GC/MS measurement, such that the instrumental error does not obscure physiological oscillations of glycolysis. (2) Oscillatory nature of glycolysis poses a challenge for statistical comparison of glycolytic intermediates, because the distribution is not governed by random error and therefore is likely to deviate from the normal distribution. In fact, three analytes in the Control group, G1-P, G6-P, and F6-P, fail the normality test (see S1 Table). In case of pyruvate in the Control group, the probability of the distribution being normal is low, only 6.7%. Because the tests for normality generally suffer from low power especially for small samples (i.e., these tests have relatively high probability of type II error), it is possible that with a larger number of observations more glycolytic intermediates would show a significant deviation from normal distribution.

Clearly, even if only the Control group deviates from normal distribution, the ANOVA test cannot be used to compare Control, SHF and DHF. It is common to use parametric tests (i.e., t-test and ANOVA) for statistical analysis of massive –omics data without testing for normality. For example, Metaboanalyst web-based service is popular and extremely useful to analyze a large set of metabolites[3,4]; however, it does not offer non-parametric tests for comparisons between groups. The analysis presented here urges caution regarding analysis of glycolytic intermediates. This may also be relevant to other metabolites which undergo periodic oscillations in living systems. If the amplitude of oscillations is larger than noise, the distribution is likely to deviate from normal. For example, the probability distribution of the sine wave is U-shaped, as opposed to the bell-shaped normal distribution.

The ideal statistical test for assessment of oscillating processes would be based on the known probability distribution for the particular type of oscillation. We are not aware of any mathematical apparatus, which could be applied to random samples of glycolytic intermediates taking into account the oscillatory nature of glycolysis. Clearly, development of such an apparatus would be beneficial for metabolomics analysis.

Considering practical approach for the current study, we believe that a nonparametric test should be used for analysis of glycolytic intermediates. S1 Table compares the results obtained by parametric (ANOVA and Fisher’s post-hoc test) versus non-parametric (Kruskal-Wallis and Dunn post-hoc test) statistical analysis. It is clear that the non-parametric analysis reveals more significant differences than parametric analysis. For SHF, the non-parametric tests indicate that all intermediates except phosphoenolpyruvate are significantly reduced as compared to Control. For DHF, only G6-P, phosphoenolpyruvate, and lactate are different from Control. However, since the pattern of change in the distributions of individual glycolytic intermediates is similar between DHF and SHF (see S4 Fig), we believe that other intermediates are also reduced in DHF as compared to control, but the power of the statistical test used is not sufficient to ascertain the difference.

Importantly, however, the test reveals a significant decrease in G6-P in both HF groups without a decrease in tissue glucose, suggesting that the rate-limiting step of glycolysis is at the level of hexokinase in our HF model. Together with a dramatic decrease in lactate in both HF models (confirmed in DHF by an enzymatic assay, see S6 Fig), these data suggest that glucose oxidation is substrate-limited (see more in the Discussion of the main text).

**2-Aminoadipic acid – a biomarker of HF?**

It is important to note that the unbiased metabolomic screening of myocardial tissue led us to discover 2-aminoadipic acid (2-AAA) as a biomarker of HF. The myocardial level of 2-AAA was significantly decreased in both failing hearts (S8 Fig. Panel A). Moreover, it appears that the level of myocardial 2-AAA was correlated to LV dysfunction (Control>SHF>DHF). Although 2-AAA is a poorly characterized product of lysine degradation, the plasma level of 2-AAA became a new biomarker of diabetes, which was also discovered using metabolomic technique[5]. However, in diabetic patients the level of plasma 2-AAA in plasma and skin tissue increased in patients, possibly due to the high blood glucose. Since we did not see the increased blood glucose in post-paced animals (S8 Fig. Panel B), it is unlikely that the difference in the level of myocardial 2-AAA was associated with diabetes. Thus, 2-AAA may be a new myocardial biomarker of HF and an indicator of LV dysfunction.

**Na^+^/K^+^ pump**

Through our proteomic screening, we detected ∂- and ß-subunits of Na^+^/K^+^ pump, which plays an essential role in maintaining Na^+^-gradient across the membrane. Interestingly, the expression level of Na^+^/K^+^ pump ß-subunit, which is important for the integration of the pump in the membrane, significantly reduced in DHF as compared to Control and SHF (S9 Fig). It is plausible that downregulation of Na^+^/K^+^ pump disrupted Na^+^-gradient in DHF. Indeed, it has been reported that intracellular Na^+^ concentration elevates in failing hearts [6,7]. It is interesting to note that cellular uptake of creatine and carnitine is mediated by Na^+^-dependent transporters (CrT and OCTN2, respectively, [8,9]) and that the myocardial levels of both creatine and carnitine significantly decreased in DHF. Thus, downregulation of Na^+^/K^+^ pump in DHF might indicate a link between metabolic and electrophysiological remodeling, which needs to be further elucidated.

**References (S1 Text)**

1. Richter PH, Ross J (1980) Oscillations and efficiency in glycolysis. Biophys Chem 12: 285-297.

2. Betz A, Chance B (1965) Phase Relationship of Glycolytic Intermediates in Yeast Cells with Oscillatory Metabolic Control. Arch Biochem Biophys 109: 585-594.

3. Xia J, Psychogios N, Young N, Wishart DS (2009) MetaboAnalyst: a web server for metabolomic data analysis and interpretation. Nucleic Acids Res 37: W652-660.

4. Xia J, Mandal R, Sinelnikov IV, Broadhurst D, Wishart DS (2012) MetaboAnalyst 2.0--a comprehensive server for metabolomic data analysis. Nucleic Acids Res 40: W127-133.

5. Wang TJ, Ngo D, Psychogios N, Dejam A, Larson MG, et al. (2013) 2-Aminoadipic acid is a biomarker for diabetes risk. J Clin Invest 123: 4309-4317.

6. Pogwizd SM, Sipido KR, Verdonck F, Bers DM (2003) Intracellular Na in animal models of hypertrophy and heart failure: contractile function and arrhythmogenesis. Cardiovasc Res 57: 887-896.

7. Bay J, Kohlhaas M, Maack C (2013) Intracellular Na(+) and cardiac metabolism. J Mol Cell Cardiol 61: 20-27.

8. Vary TC, Neely JR (1983) Sodium dependence of carnitine transport in isolated perfused adult rat hearts. Am J Physiol 244: H247-252.

9. Peral MJ, Vazquez-Carretero MD, Ilundain AA (2010) Na(+)/Cl(-)/creatine transporter activity and expression in rat brain synaptosomes. Neuroscience 165: 53-60.

10. Shen W, Asai K, Uechi M, Mathier MA, Shannon RP, et al. (1999) Progressive loss of myocardial ATP due to a loss of total purines during the development of heart failure in dogs: a compensatory role for the parallel loss of creatine. Circulation 100: 2113-2118.
